# Discovery of a novel emaravirus and an alphacytorhabdovirus infecting *Spiraea* in the USA

**DOI:** 10.1007/s00705-026-06640-2

**Published:** 2026-06-11

**Authors:** Joana Serrano Salgado, Milenka Vera, Ronan Keener, Jackson Gray, Brett Arenz, Dimitre Mollov, Pavel B. Kilmov, Robert Alvarez-Quinto

**Affiliations:** 1 Department of Plant Pathology, University of Minnesota, Saint Paul, MN 55108 USA; 2 Pest Exclusion and Import Programs, USDA APHIS Plant Protection and Quarantine, Beltsville, MD 20705 USA; 3 Department of Biological Sciences, Purdue University, 915 Mitch Daniels Blvd, West Lafayette, IN 47907 USA

## Abstract

Spiraea plants showing symptoms of virus infection were investigated using high-throughput sequencing which resulted in the identification of two novel viruses: an emaravirus named Spiraea chlorotic leaf spot distortion virus (SCLSDV), and an alphacytorhabdovirus named Spiraea alphacytorhabdovirus 1 (SpCRV-1). The complete genome of SCLSDV comprises four RNA segments, each encoding a distinct protein: RNA-dependent RNA polymerase, glycoprotein precursor, nucleocapsid protein, and movement protein, respectively. SCLSDV shares 33.99–71.78% amino acid identity with pear chlorotic leaf spot-associated virus. Virus purification and electron microscopy confirmed pleomorphic, enveloped particles (80–100 nm) matching the morphology of members of the genus *Emaravirus*. The alphacytorhabdovirus shares 70% of nucleotide identity with Actinidia virus D and encodes eight putative proteins. Both novel viruses were detected in a mixed infection with Spiraea yellow leaf spot virus. Additionally, SCLSDV was consistently detected in a single infection in 50 out of 77 plant samples exhibiting yellow spot disease, characterized by chlorotic lesions, leaf distortion, and necrosis. These findings suggest that strong yellow spot symptoms and leaf distortion are likely associated with SCLSDV infection. Furthermore, eriophyid mites were collected from symptomatic plants and identified morphologically as *Calepitrimerus* sp. Given the known role of eriophyid mites in emaravirus transmission, their presence suggests it could be a potential vector for SCLSDV.

**Supplementary Information:**

The online version contains supplementary material available at 10.1007/s00705-026-06640-2.

## Introduction

*Spiraea* spp. (family: Rosacea, Genus: *Spiraea*) are native to the temperate northern hemisphere and are widely cultivated ornamental shrubs. Four viruses have been reported in spiraeas: (i) plum pox virus (PPV, *Potyvirus*), (ii) Arabis mosaic virus (ArMV, *Nepovirus*), (iii) Spiraea yellow leaf spot virus (SYLSV, *Badnavirus*) and (iv) Spiraea yellow spot spherical virus (SYLSSV, Totivirus-like, unclassified) [32, 41]. In the USA, SYLSV and SYLSSV were found to be associated with yellow spot disease of spiraea [2, 32].

Even though yellow spot disease of spiraea has been commonly associated with SYLSV and SYLSSV, our recent diagnostic efforts have resulted in inconsistencies between yellow spot disease symptoms and known viral pathogens. Beginning in 2024, leaf samples of spiraea from commercial nurseries in five U.S. states (Indiana, Minnesota, New York, Ohio, Oregon) were submitted for virus testing at the University of Minnesota (UMN) Plant Disease Clinic showing symptoms suggestive of viral infection, but tested negative for the previously reported spirea viruses. Some symptomatic plants also generally had abundant eriophyid mite infestations, and in some cases this was previously assumed to be the cause of the symptoms in some situations. Upon analysis with high-throughput sequencing (HTS) it was found that these symptomatic plants contained previously uncharacterized viruses, which the mites were likely to be serving as vectors. This paper describes the characterization of a novel emaravirus and alphacytorhabdovirus infecting spiraea shrubs in the USA.

The genus *Emaravirus* is classified in the family *Fimoviridae* (order *Elliovirales*) and includes 33 species as determined by the International Committee on Taxonomy of Viruses (ICTV) [2]. This genus is characterized by virus species with negative sense multipartite single-stranded RNA (ssRNA) genome, consisting of 4 to 10 segments with high genome plasticity. At least four core segments are involved in replication, virion assembly, and movement (RNA 1 - RNA 4), with potentially more segments (non-core segments) either by reassortment or duplication to promote other functions as pathogenicity, silencing suppression, and host range [11, 21, 33]. The 5’ and 3’ ends of all RNA segments have partial complementary, prone to constructing panhandle structures [4, 5, 7, 8]. Virions are pleomorphic spherical enveloped particles with a diameter size of 80–100 nm enclosed in a double-membrane envelope [11, 39, 43, 45]. Emaraviruses infect a broad host range and can be transmitted by grafting, mechanical inoculation, and eriophyid mites [12, 27, 29, 30, 38, 45, 56].

The family *Rhabdoviridae* encompasses a large group of negative-stranded RNA viruses that infect a wide variety of hosts, including mammals, birds, reptiles, fish, invertebrates (arthropods), and plants [6]. Cytorhabdoviruses replicate and mature in the cytoplasm of the infected host cells, where virioplasms accumulate [25]. Current taxonomic and phylogenetic analyses have shown that cytorhabdoviruses are split into three separate genera: *Alphacytorhabdovirus*,* Betacytorhabdovirus and Gammacytorhabdovirus* within the family *Rhabdoviridae* [9].

## Materials and methods

### Virus source

A total of 77 spiraea samples were collected from several locations in Minnesota, including the MN Landscape Arboretum and the Ordway Japanese Garden. Additional samples were received for diagnostics at the UMN Plant Disease Clinic from Indiana, New York, Ohio, and Oregon (Supplementary Table S1).

### High-throughput sequencing and bioinformatics

Total nucleic acids (TNA) were extracted from ∼100 mg of leaf tissue using a lithium chloride and potassium acetate based protocol [58]. Silica glass milk (Silicon dioxide) was used for nucleic acid capture [22]. TNA was DNase-treated and used as a template to prepare an RNA-Seq library using the TruSeq Stranded Total RNA with Ribo-Zero Plant kit (Illumina). A total of four samples (SPR621, SP2019, SPWSTP, SPR-OR6) from Minnesota and Oregon were sequenced as 150 paired-end reads on a NovaSeqX platform (Psomagen). Raw sequence data were pre-processed with the BBDuk adapter/quality trimming tool version 38.84. Trimmed sequences were assembled using SPAdes assembler 3.15.2 and Trinity Release-v2.15.2. Viral contigs were identified by BLASTx search (NCBI) using the high-performance computing (HPC) resources of the Minnesota Supercomputer Institute (MSI). Additionally, reference-guided assembly was carried out using BBMap version 1.0. The identification of open reading frames (ORFs), conserved and functional domains, transmembrane helices, N-glycosylation sites, signal peptides, cleavage sites, and panhandle structures of the terminal ends was predicted using the NCBI tools ORFinder (NCBI), Conserved Domain Database (CDD) Search tool [36], SMART tool in genomic mode [31], Deep TMHMM [23], NetNGlyc − 1.0 [20], SignalP − 6.0 [52], and RNAfold web server [19], respectively.

### Rapid amplification of cDNA ends (RACE)

The full-length genomic sequences of the new emaravirus isolate SPR621 were determined, and the termini regions of each RNA segment were obtained using a 5’/3’ RACE Kit, Second Generation (Roche, Germany). For the 3’ end, the RNA was polyadenylated with PolyA polymerase (NEB, USA). TNA was mixed with 10% DMSO, incubated at 98 °C for 10 min, and then placed on ice for two minutes before polyA tailing and reverse transcription. Several primers were used to determine the terminal regions (Supplementary Table S2). RT-PCR amplicons were cloned using TOPO™ XL-2 PCR Cloning Kit, five clones per cloning reaction were sequenced in both directions by Sanger sequencing at MCLAB (California, USA).

### Virus detection

TNA was extracted from ~ 100 mg of plant tissue, as described above [22]. Reverse transcription (RT) was done in a 10 µL reaction mixture containing 2 µL of 5X SuperScript IV Buffer, 0.5 µL of random primers 40 µM, 0.5 uL of 100 mM DTT, 0.5 µL of 10 mM dNTP mix, 0.1 µL of SuperScript IV reverse transcriptase (Thermo Fisher), 0.1 µL of RNAse Inhibitor, and 2 µL (∼150 ng) of TNA template.

RT reactions were incubated at 55 °C for 20 min and then at 80 °C for 10 min. PCR was done in 10 µL reactions using 5 µL of 2X GoTaq Green Master mix (Promega, Madison, WI, U.S.A.), 2 µL of nuclease-free water, 1 µL of cDNA, and 1 µL of each primer at 5 µM. Virus-specific primers were designed based on conserved regions identified from the HTS assemblies (Table 1). A fragment of 181 bp length, corresponding to the plant NAD5 (dehydrogenase ND2 subunit), was amplified as the internal control [37]. PCR amplification parameters were as follows: an initial denaturation step at 95 °C for 2 min followed by 40 cycles of 95 °C for 30 s, 55 °C for 30 s, and 72 °C for 40 s, and a final extension at 72 °C for 5 min.

**Table 1:** Detection primers for reverse-transcription PCR tests of Spiraea chlorotic leaf spot distortion virus (SCLSDV), Spiraea alphacytorhabdovirus 1 (SpCRV-1), Spiraea yellow spot virus (SYLSV), and internal control *nad* 5

Primer name	Sequence 5’ to 3’	RNA - Target	Annealing temperature (°C)	Expected size (bp)	Source
EV NCF1	TGCCCTGACAGTATCGAGCT	RNA 3 - Nucleocapsid	55	384	This study
EV NCR1	AGGGATGCGACTGTCGAGTT				
EV Rep F1	GAGCATCTTCTATTGTGTTGTGTGC	RNA 1 - Replicase	55	861	
EV Rep R1	GAGAAAGGGGTGCTTTATCTGTTTC				
SPCR_NC_R1	TCACTCTGTGTACCATATGGAGCC	Nucleocapsid	55	491	This study
SPCR_NC_F1	CCCGTCACTGTCGGATTATTGAGG				
SYLSV Det F1	CCAAATCGCTCGAGCAGAAGC	ORF3 - Polyprotein	57	529	(Alvarez-Quinto et al. 2022)
SYLSV Det R1	CCATCGACAGCTATCGAATCTGC				
Nad5-F	GATGCTTCTTGGGGCTTCTTGTT	Nad5 - Internal control ^a^	55	181	(Menzel et al. 2002)
Nad5-R	CTCCAGTCACCAACATTGGCATAA				

^a^ NADH dehydrogenase 5

### Phylogenetic analysis

The genetic sequences corresponding to the RNA-dependent RNA polymerase (RdRp), glycoprotein precursor (GP), nucleocapsid (NC), and movement protein (MP) of accepted virus species in the genus *Emaravirus*, as well as the polymerase gene (L) of accepted virus species within the genera *Alphacytorhabdovirus*,* Betacytorhabdovirus*,* and Gammacytorhabdovirus*, were retrieved from GenBank using Batch Entrez (NCBI).

Sequences were translated and then aligned using MAFFT version 7. The best amino acid partition model for each protein sequence was determined using MEGA X. Maximum Likelihood (ML) analysis was performed using RAxML v8.2.11 utilizing the AVX-optimized binaries. ML trees were inferred from 1,000 bootstrap replicates. These analyses were performed on the HPC resources of the MSI.

### Virus purification and transmission electron microscopy visualization

Virus-like particles were purified according to Tsuda et al., 1991 [53] with some modifications. Twelve grams of leaf tissue was ground with liquid nitrogen and extracted with 1:6 (w/v) 0.1 M NaPO4 pH 7.0, 0.01 M sodium sulfite, and 0.01 M EDTA. The extract was filtered through cheesecloth and centrifuged at 10,000 g_max_ for 15 min with Beckman JA-17 rotor. The supernatant was layered over 5 mL 25% sucrose and centrifuged at 235,000 g_max_ for 1 h 30 min using Beckman type 50.2Ti. The pellet was resuspended in 8 mL of resuspension buffer (0.1 M NaPO_4_ pH 7.0, 0.5 M EDTA pH 7.0, 500 mg of L-cysteine) and centrifuged at 10,000 g_max_ for 10 min using Beckman JA-17 rotor. The supernatant was layered over 5 ml 25% sucrose and centrifuged at 235,000 g_max_ for 1 h 30 min using a Beckman type 50.2Ti rotor and polycarbonate tubes. The pellet was resuspended with 1mL of resuspension buffer, layered over a stepwise gradient of 15, 40, and 50% sucrose and centrifuged at 113,000 g_max_ for 16 h using a Beckman SW28 rotor. Fractions of 500 µL were collected and screened by RT-PCR. Subsequently, four fractions corresponding to the virus band (where light diffraction was observed) and intense RT-PCR amplicon signal were consolidated and centrifuged at 214,200 g_max_ for 1 h 30 min using a Beckman type 90Ti rotor. The final pellet was resuspended with 100 µL of resuspension buffer and left at 4 °C for 10 min. Virus suspension was mounted on a carbon-coated formvar grid and negatively stained with 2% Phosphotungstic acid. Visualization was done at the University of Minnesota - University Imaging Center (UIC) using JEOL JEM-1400Plus transmission electron microscope (TEM).

### Mites mounting and characterization

Infested plant samples were photographed before mounting in the laboratory using an Olympus EP50 camera attached to an Olympus SZ61 dissecting microscope. Mites were collected by alcohol wash. Samples were preserved in 1.5 mL microcentrifuge tubes filled with 95% ethanol and stored at -80 °C. For maceration and clearing, 0.5–1 mL of 30% lactic acid was added, and the samples were incubated at 50 °C for 12–24 h in a three-well glass plate to remove highly refractive guanine crystals inside mites. Mites then were mounted in Hoyer’s medium on microscope slides and dried at 50 °C for one week. Mites were identified and photographed under a Zeiss Imager.M2 compound microscope equipped with 100× objectives, DIC optics, and a Zeiss Axiocam 820 color camera. Slides are preserved at Purdue University under lot accession number PBK-22-0905-304.

## Results

### Identification of viruses by HTS

HTS analysis of symptomatic spirea samples revealed the presence of viral contigs corresponding to two distinct groups of negative-sense RNA viruses. A set of contigs revealed homology to the four core RNA segments of members of the genus *Emaravirus*, with amino acid identities ranging from 33.99% to 71.68% relative to pear chlorotic leaf spot associated virus (PCLSaV). In addition, near-complete genomes sharing 73% nt identity to Actinidia virus D (AcVD, *Alphacytorhabdovirus*) were detected in samples SP2019 (Minnesota) and SPR-OR6 (Oregon). Additionally, a full-length viral sequence of Spiraea yellow leaf spot virus (SYLSV, *Badnavirus*) was assembled from sample SP2019, showing 98.04% nt identity to the SYLSV isolate MD (MW080370). The detection of SYLSV indicates the occurrence of mixed infections in spiraea. A complete summary of the results is presented in Table 2.

**Table 2:** Summary of viral contigs assembled from high-throughput sequencing (HTS) data on contig length, BLASTx homology (genus and genomic segment), sequence identity (ID%) at the amino acid level, number of reads in the contig, mean coverage (x) per nucleotide, and GenBank accession numbers

Sample	Total reads	Contig length (nt)	Blastx homology (Genus) - Genomic Segments	Closest hit (Accession number)	ID % (aa)	No. of reads in contig (%) ^a^	Mean coverage (X per nt)	Accession number (NCBI, GenBank)
SPR621	59,625,764	7,145	Pear chlorotic leaf spot-associated virus (*Emaravirus*) - RNA 1 - RdRp	QKY76998	54.02	51,851 (0.09)	1,071.6	PV054127
		2,028	Pear chlorotic leaf spot-associated virus (*Emaravirus*) - RNA 2 - Glycoprotein	YP_010840090	38.45	5,412 (0.01)	437.4	PV054128
		1,217	Pear chlorotic leaf spot-associated virus (*Emaravirus*) - RNA 3 - Nucleocapsid protein	YP_010840086	33.99	47,668 (0.08)	7,217.6	PV054129
		1,470	Pear chlorotic leaf spot-associated virus (*Emaravirus*) - RNA 4 - Movement protein	QKY77001	71.68	15,695 (0.03)	2,179.7	PV054130
SP2019	40,826,964	7096	Pear chlorotic leaf spot-associated virus (*Emaravirus*) - RNA 1 - RdRp	QKY76998	54.18	39,598 (0.10)	800.4	PV091816
		7093	Pear chlorotic leaf spot-associated virus (*Emaravirus*) - RNA 1 - RdRp	QKY76998	54.09	8,362 (0.02)	170	PV091817
		2007	Pear chlorotic leaf spot-associated virus (*Emaravirus*) - RNA 2 - Glycoprotein	YP_010840090	38.28	2,174 (0.01)	172.7	PV091821
		2018	Pear chlorotic leaf spot-associated virus (*Emaravirus*) - RNA 2 - Glycoprotein	BCM23429	37.93	10,322 (0.03)	801.9	PV091822
		1373	Pear chlorotic leaf spot-associated virus (*Emaravirus*) - RNA 3 - Nucleocapsid protein	YP_010840086	33.99	20,358 (0.05)	2753.7	PV091825
		1366	Pear chlorotic leaf spot-associated virus (*Emaravirus*) - RNA 3 - Nucleocapsid protein	YP_010840086	33.99	32,919 (0.1)	4456.7	PV091826
		1052	Pear chlorotic leaf spot-associated virus (*Emaravirus*) - RNA 3 - Nucleocapsid protein	YP_010840086	33.99	7,801 (0.02)	1621.6	PV091827
		1475	Pear chlorotic leaf spot-associated virus (*Emaravirus*) - RNA 4 - Movement protein	QKY77001	71.68	19,149 (0.05)	2111.8	PV091832
		8,020	Spiraea yellow leafspot virus (*Badnavirus*) - ORF3	QVX19934	97.95	7,201 (0.02)	127.5	PV235597
		13,629	Actinidia cytorhabdovirus JS27 (*Alphacytorhabdovirus*) - RdRp	YP_010805017	83.86	1,690 (0.004)	17	PV231214
SPWSTP	43,115,622	7126	Pear chlorotic leaf spot-associated virus (*Emaravirus*) - RNA 1 - RdRp	QKY76998	54.11	33,523 (0.08)	707.4	PV091814
		7073	Pear chlorotic leaf spot-associated virus (*Emaravirus*) - RNA 1 - RdRp	QKY76998	54.18	8,962 (0.02)	186.4	PV091815
		2053	Pear chlorotic leaf spot-associated virus (*Emaravirus*) - RNA 2 - Glycoprotein	YP_010840090	38.45	976 (0.002)	72.2	PV091819
		2048	Pear chlorotic leaf spot-associated virus (*Emaravirus*) - RNA 2 - Glycoprotein	YP_010840090	38.45	4,750 (0.01)	408.4	PV091820
		1182	Pear chlorotic leaf spot-associated virus (*Emaravirus*) - RNA 3 - Nucleocapsid protein	YP_010840086	33.99	38,206 (0.09)	5676.5	PV091824
		1517	Pear chlorotic leaf spot-associated virus (*Emaravirus*) - RNA 4 - Movement protein	QKY77001	71.78	3,780 (0.01)	437	PV091830
		1513	Pear chlorotic leaf spot-associated virus (*Emaravirus*) - RNA 4 - Movement protein	QKY77001	71.78	3,583 (0.01)	449.4	PV091831
SPR-OR6	52,339,914	7086	Pear chlorotic leaf spot-associated virus (*Emaravirus*) - RNA 1 - RdRp	QKY76998	54.02	92,200 (0.18)	1956.4	PV091818
		2039	Pear chlorotic leaf spot-associated virus (*Emaravirus*) - RNA 2 - Glycoprotein	YP_010840090	37.59	20,363 (0.04)	1899.9	PV091823
		1362	Pear chlorotic leaf spot-associated virus (*Emaravirus*) - RNA 3 - Nucleocapsid protein	YP_010840086	33.99	51,480 (0.10)	7469.6	PV091828
		1150	Pear chlorotic leaf spot-associated virus (*Emaravirus*) - RNA 3 - Nucleocapsid protein	YP_010840086	33.99	50,794 (0.10)	7919.1	PV091829
		1476	Pear chlorotic leaf spot-associated virus (*Emaravirus*) - RNA 4 - Movement protein	QKY77001	71.68	44,564 (0.09)	6056.0	PV091833
		13,628	Actinidia cytorhabdovirus JS27 (*Alphacytorhabdovirus*) - RdRp	YP_010805017	84.26	1,230 (0.002)	12.5	PV231215

^a^ Percentage of reads per contig is given relative to the total number of reads in each sample

### Genome assembly, organization, and phylogeny of the novel emaravirus

**Fig. 1:**
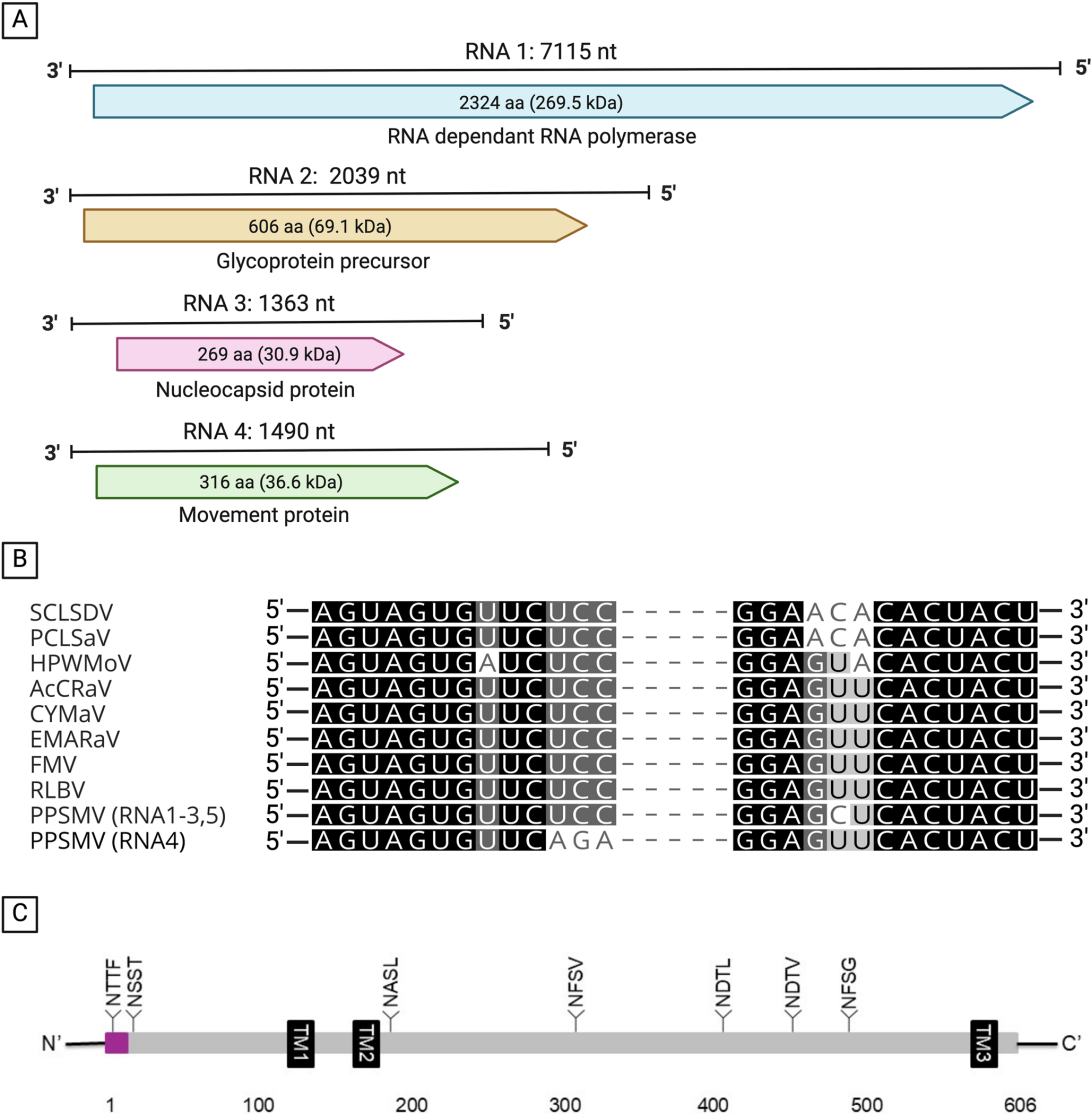
Genome organization and proteins of Spiraea chlorotic leaf spot distortion virus (SCLSDV). (**A**) Diagram of the genome organization. Expression product of each RNA is shown as a different color and the potential function of the putative protein is noted. (**B**) Pairwise alignment of the 13 nt conserved stretches located at each termini. Approximate size scale (kb) is shown below the RNA genome. (**C**) Conserved transmembrane domains (TMs) of hypothetical glycoprotein precursor sequence are indicated by black rectangles, signal peptide by a purple box, and seven putative glycosylation sites (NTTF, NSST, NASL, NFSV, NDTL, NDTV, NFSG) by the “Y” symbol

The complete genome of the putative emaravirus was fully sequenced from sample SPR621 (West Saint Paul, Minnesota) through RACE amplification. The complete genome of this emaravirus consists of four genomic RNAs segments, each encoding a single ORF on their complementary-sense RNA (Fig. 1A). All four genomic RNA segments share a conserved 13 nt motif at the 5’ terminus (3’-AGUAGUGUUCUCC-5’) and 3’ terminus (3’-GGAGAACACUACU-5’) ends, which is identical to the terminal sequence of PCLSaV (Fig. 1B). The 5’ and 3’ untranslated regions (UTRs) of each genomic RNAs were predicted to form a complementary base-paired RNA panhandle structure (Supplementary Fig. S1) similar to that described in the order *Elliovirales* [9, 13, 26].

RNA 1 encodes a putative RdRp (Table 2). This protein contains a conserved domain within the bunyavirus RdRp superfamily and includes motifs characteristic of emaraviruses (Supplementary Fig. S3A). Motifs A and C are part of the palm domain involved in divalent cation binding [10, 34], while motif B is expected to have RNA binding activity [30]. Additionally, the N-terminus of RdRp contains a conserved endonuclease domain (Supplementary Fig. S4), likely involved in the “cap-snatching” mechanism for transcription initiation [13, 16, 44, 60].

RNA 2 encodes a putative glycoprotein precursor (GP) sharing 38.45% of amino acid identity of with PCLSaV. Predicted features include one N signal peptide, three predicted transmembrane domains (121 to 143, 169 to 187, 562 to 584 aa), and seven putative N-glycosylation sites (Fig. 1C). Unexpectedly, SignalP could not identify potential cleavage sites, and was found to lack the tetrapeptide sequence (ADDN), which has been previously described in other emaraviruses as required for cleaving the precursor into two distinct glycoproteins [43]. Similarly, SCLSDV does not contain the characteristic phlebovirus glycoprotein motif that has been documented in other emaraviruses [12, 24, 30].

RNA 3 encodes a NC sharing a 33.99% aa identity with pear chlorotic spot-associated virus. This protein comprises three amino acid stretches identified as putative emaravirus nucleocapsid motifs FVLSFGRN_111−116_ NRLA_163−166_ GMEN_184−187_ (Supplementary Fig. S5) [17, 24, 60]. The first motif exhibits significant divergence from the RNA 3 coding sequence of other emaraviruses, with only the serine (S) and arginine (R) residues conserved across all closely related emaraviruses. In contrast, the second motif is completely conserved across all emaraviruses. The final motif contains a methionine (M) at the second position, which is the only amino acid that differs from those found in other emaraviruses [28, 43, 59].

RNA 4 encodes a putative MP sharing the highest amino acid identity of 71.68% with pear chlorotic spot-associated virus. Further examination revealed the presence of a signal peptide (1–18 aa) and coiled coil sequence (279–304 aa), which are characteristic of emaravirus movement proteins [60]. However, RNA 4 does not contain the 30 kDa movement superfamily domain according to CDD results.

**Fig. 2:**
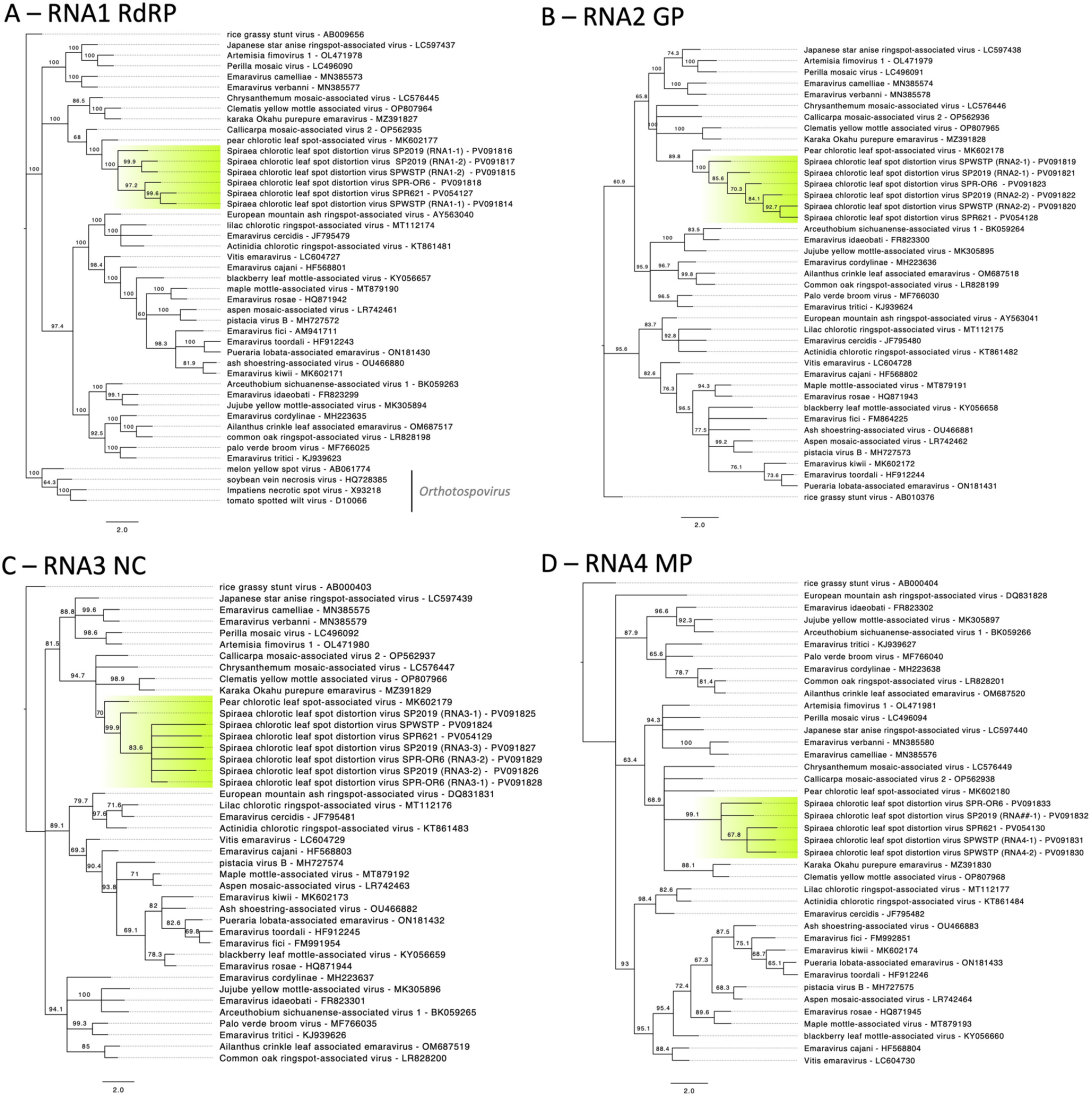
Maximum-likelihood (ML) phylogenetic trees generated from the deduced amino acid sequences of the (**A**) RNA-dependent RNA polymerase (RNA1 - RdRP), (**B**) glycoprotein precursor (RNA2 -GP), (**C**) nucleocapsid protein (RNA3 - NC), (**D**) movement protein (RNA4 - MP) of emaraviruses. GenBank accession numbers of proteins applied for phylogenetic evaluation are listed alongside the complete name of each virus. Spiraea chlorotic leaf spot distortion virus (SCLSDV) is highlighted in green. Bootstrap values are indicated in the nodes. Rice grassy stunt virus (genus *Tenuivirus*) was used for outgroups in each RNA

Average sequence depth across all four genomic RNAs were 1,197X, 538X, 7,773X, and 2,217X per nucleotide, respectively (Supplementary Fig. S2A–S2D). Maximum likelihood phylogenetic analyses of the putative RdRp, GP, NC, and MP of the spiraea emaravirus consistently placed it within members of the genus *Emaravirus*, sharing a common ancestor with PCLSaV, CMaV2, ChMaV, CYMaV, KOPV (Fig. 2).

The RdRp, GP, and NP proteins of the spiraea emaravirus display significant divergence from known emaraviruses. Additionally, the genome structure of the spiraea emaravirus, conserved terminal sequences, and functional motifs are consistent with members of the genus *Emaravirus* and support our taxonomic placement. Following the ICTV demarcation criteria based on differences more than 25% in the amino acid sequences of relevant gene products RdRp, GP, NP; we propose that the spiraea emaravirus should be considered a distinct virus species. We suggest the name Spiraea chlorotic leaf spot distortion virus (SCLSDV), which we will use hereafter. The complete genome sequence of SCLSDV isolate SPR621 was submitted to the NCBI under the accession numbers PV054127-30.

### Genome assembly, organization, and phylogeny of the novel alphacytorhabdovirus

**Fig. 3:**
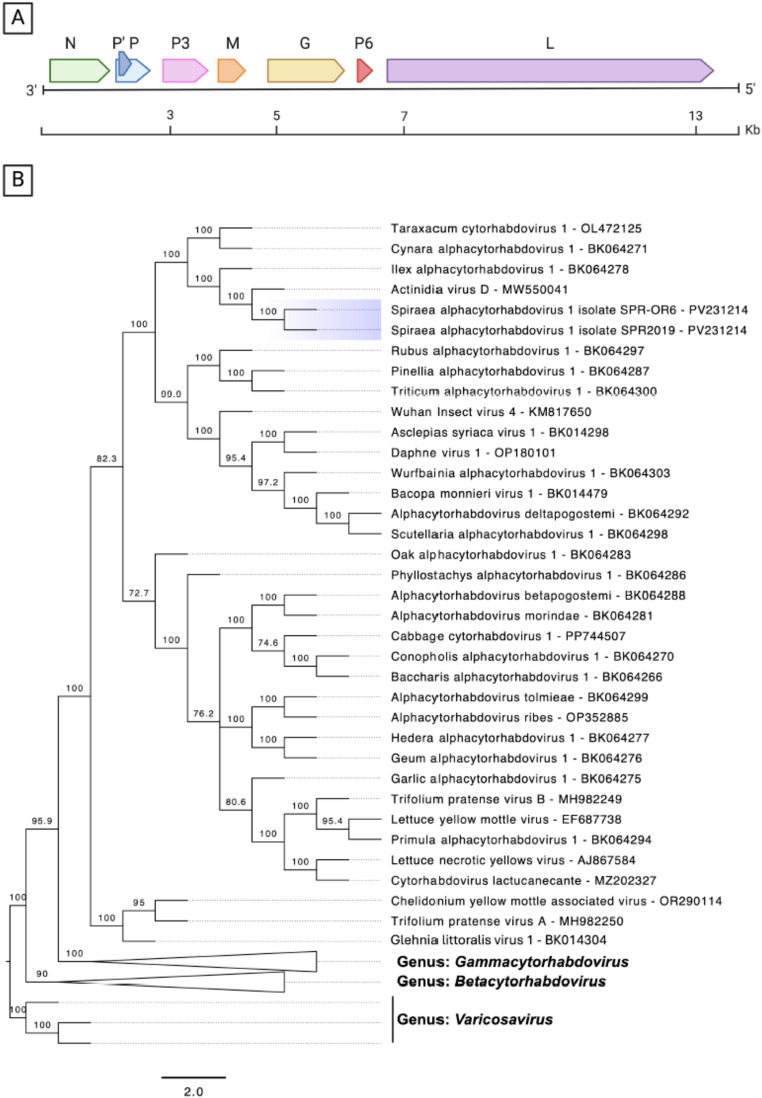
Genome organization and phylogenetic analysis of Spiraea alphacytorhabdovirus 1 (SpCRV-1). (**A**) Graphic diagram of genome organization. Open reading frames (ORFs) are represented by color and arrowed rectangle figures, and the encoded protein is labeled above each ORF. The approximate size scale (kb) is shown below the RNA genome. (**B**) Maximum-likelihood (ML) phylogenetic trees generated from the deduced amino acid sequences corresponding to the replicase of the alphacytorhabdovirus (SP2019 and SPR-OR6) and selected members of the genera *Alphacytorhabdovirus*, *Betacytorhabdovirus*, and *Gammacytorhabdovirus*. GenBank accession numbers of the sequences used for the phylogenetic analysis are listed alongside the complete name of each virus. Spiraea alphacytorhabdovirus 1 are highlighted in purple. Bootstrap values are indicated in the nodes. Lettuce big-vein virus, red clover varicosavirus, and black grass varicosavirus (genus *Varicosavirus*) were used for outgroups

Two near-complete alphacytorhabdoviral genomes were assembled from samples SP2019 and SPR-OR6 (Supplementary Fig. S6A, B). BLASTn analysis identified Actinidia virus D (AcVD), as the closest relative with nucleotide identities of approximately 75%. The genome organization of this virus is consistent with that of plant infecting rhabdoviruses, displaying eight canonical ORFs with products arranged in the classical monopartite plant rhabdovirus organization: 3-leader-N-P-P’-P3(MP)-M-G-P6-L-5-trailer, with two accessory ORFs encoding hypothetical proteins (Fig. 3A).

Conserved intergenic regions (IR) 3′-AAUUAUUUUGAU(N)CU(G/U)-5′ were identified across gene junctions, exhibiting the typical three elements of Rhabdovirus IRs: a poly-U tract, intergenic spacer, and a transcription initiation signal. These IRs were highly conserved across the genome and closely resembled those of AcVD and related alphacytorhabdoviruses, with only minor sequence variation (highlighted in orange box Supplementary Fig. S7). Protein analysis revealed that the N protein belongs to the rhabdovirus nucleoprotein superfamily and contains a predicted bipartite nuclear localization signal (NLS). The P’ ORF overlaps with the phosphoprotein gene and encodes a small protein with a predicted transmembrane domain. The movement protein (P3/MP) contains a movement protein-like domain and a bipartite NLS, while the matrix protein also harbors a predicted NLS both at the C-terminal region. The glycoprotein includes a signal peptide, multiple predicted N-glycosylation sites (NTTT, NPSG, NYTN, NLSY), and a transmembrane domain, just as the P6 protein contains a predicted transmembrane region. The L protein encodes the RdRp and contains conserved domains and six motifs characteristic of mononegaviruses (Supplementary Fig. S3B). Overall, these genomic features are consistent with those described for closely related alphacytorhabdoviruses, including Actinidia virus D [57]. Key genomic features of novel alphacytorhabdovirus are summarized in Supplementary Table S3.

Pairwise amino acid sequence comparisons between the putative proteins of the new virus and those of closely related alphacytorhabdoviruses (AcVD, IleACRV1, CynACRV, TCRV1, honeysuckle-associated cytorhabdovirus 1, PinACRV1, Pelargonium radula virus 1 and Lotus corniculatus virus 1) revealed identities ranging from 38.7% to 82.8% for the nucleocapsid, 24.1% to 80.9% for the phosphoprotein, 34.7% to 87.6% for the movement protein, 13.2% to 79.4% for the matrix protein, 31.1% to 79.5% for the glycoprotein, and 45.6% to 83.8% for the polymerase (Supplementary Table S4). Following the demarcation criteria from the ICTV the alphacytorhabdvirus from spiraea meets the first criterion with nucleotide identities below 75% compared to the closest rhabdovirus. The second criterion is partially fulfilled with amino acid sequences between 79.5% − 87.6% in all cognate open reading frames to the closest alphacytorhabdovirus. Another criterion is relevant to ecological niches and host ranges and vectors.

The nucleotide identity of the genome and amino acids, along with the unique host range of the species, and phylogenetic analysis allow the virus to be classified as a new species according to the ICTV criteria for alphacytorhabdoviruses. Accordingly, we propose the name Spiraea alphacytorhabdovirus 1 (SpCRV-1). The near-complete genome sequence of SpCRV-1 isolates SP2019 and SPR-OR6 was submitted to the NCBI under the accession numbers PV231214 and PV231215. Phylogenetic analysis using the viral polymerase (L) protein revealed a well-supported clade (bootstrap value 100) containing SpCRV-1 along with AcVD, IleACRV1, CynACRV1, TRCV1 (Fig. 3B). Moreover, these four viruses exhibit a similar genomic organization.

### Virus purification and morphology

**Fig. 4:**
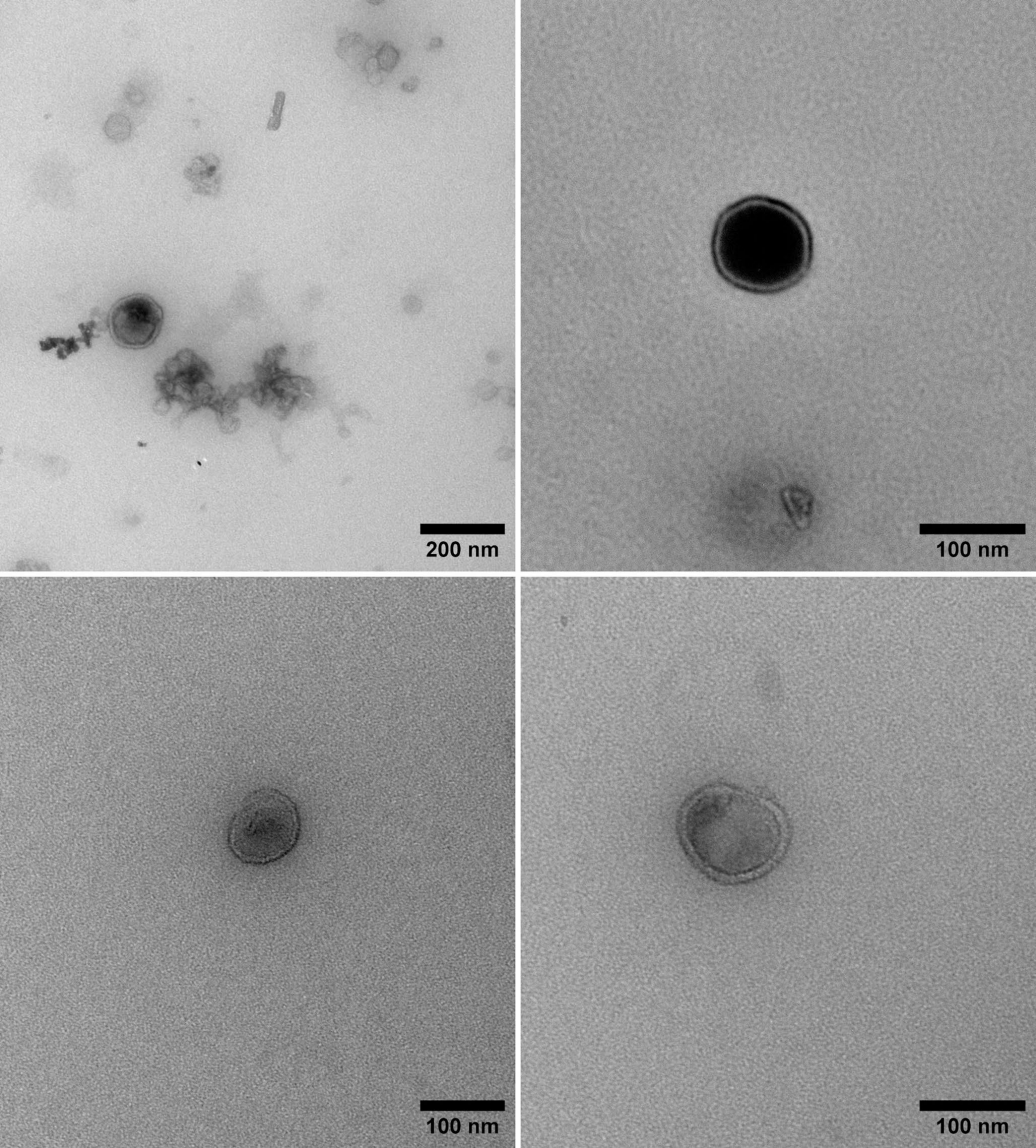
Transmission electron microscope purified virus-like particles preparation of Spiraea chlorotic leaf spot distortion virus (SCLSDV) in negative staining with 2% phosphotungstic acid showing pleomorphic enveloped particles. Scale bars represent 100 nm and 200 nm

The isolate SPR-OR-6 was utilized for purification using a stepwise sucrose gradient, and a virus band was observed. We collected fractions closest to the virus band (18, 19, 20, 21, 22, from top to bottom). TEM visualization revealed the presence of pleomorphic enveloped particles between 80 and 100 nm in diameter. As reported before, a bilipid layer was observed for members of the genus *Emaravirus* (Fig. 4). No bacilliform virus-like particles were observed.

### Occurrence of yellow spot disease symptoms and association with SCLSDV and SpCRV-1 infection

A total of 77 spiraea samples were tested for SCLSDV, SpCRV-1, and SYLSV by RT-PCR. The most prevalent virus detected was SCLSDV, with 64.94% (*n* = 50) positive samples. SpCRV-1 and SYLSV were detected at a lower rate, with 14.3% (*n* = 11) and 9.1% (*n* = 7) positive samples, respectively. SCLSDV was detected in 83.7% of samples (*n* = 43) from Minnesota and 66.7% (*n* = 9) from Oregon. Other samples from diverse origins within the USA tested at the Plant Clinic Disease were 37.5% positive (*n* = 16), including one positive sample from Ohio and another from Indiana. SpCRV-1 had the highest positive rate in samples from Oregon (44.4%, *n* = 9), followed by samples from Minnesota, with 14% (*n* = 6) in landscape ornamentals, and 6.3% (*n* = 1) received by the UMN Plant Disease Clinic. SYLSV was only detected in Minnesota in 16.3% (*n* = 43) of samples and was absent in all other locations (Supplementary Table S1).

**Fig. 5:**
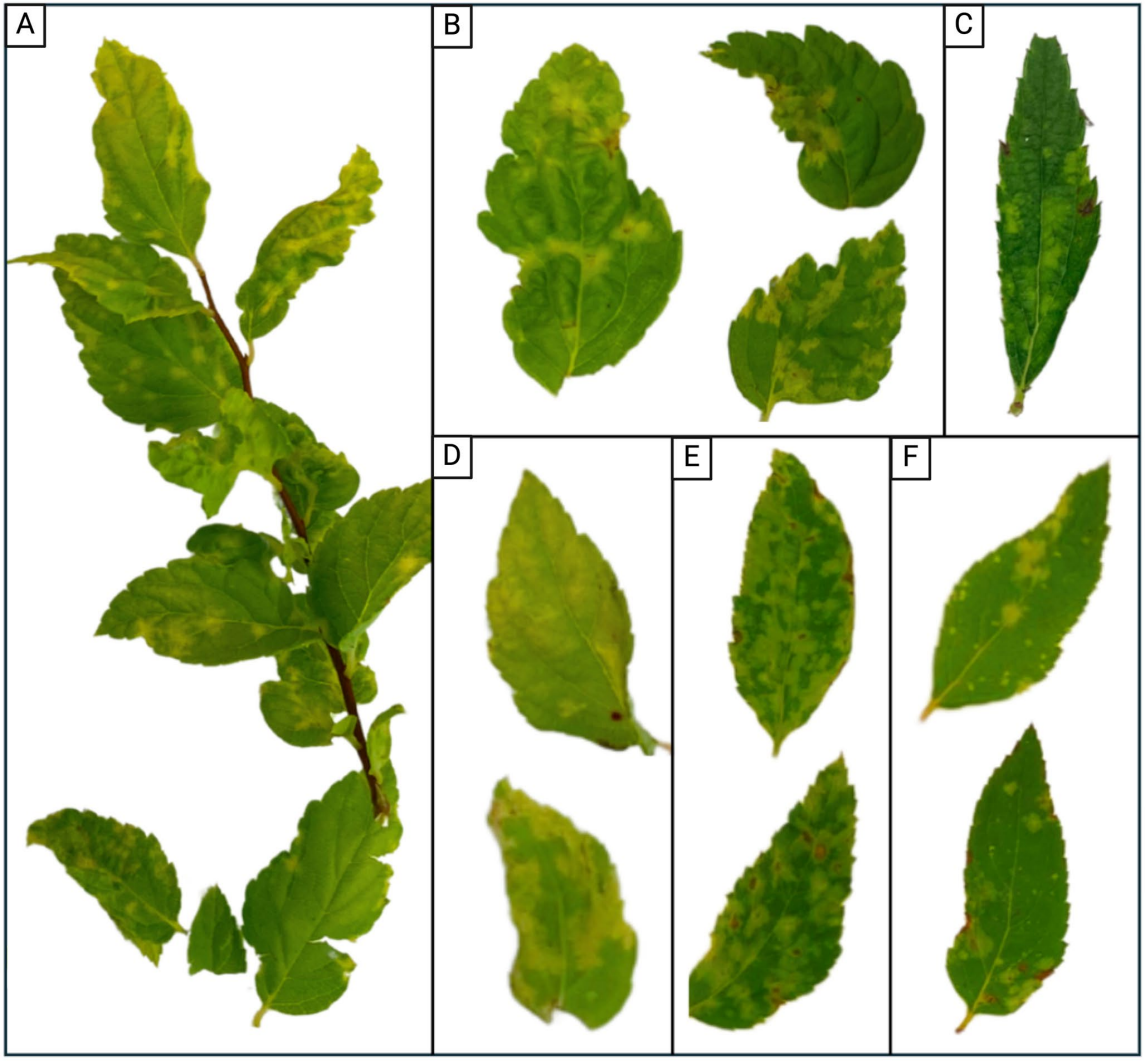
Symptoms on leaves of infected spiraeas infected by the emaravirus Spiraea chlorotic leaf spot distortion virus (SCLSDV). (**A**) yellowing, distortion, and few chlorotic spots. (**B**) Severe distortion, vein clearing, mottling, and few necrotic spots. (**C**) and F. Slight mottling. (**D**) yellowing. (**E**) severe mosaic, chlorotic spots, and few necrotic spots

In all our samples, SCLSDV was the most commonly found virus in single infection in 35 out of 77 samples (45.5%). In contrast, just two samples had a single infection of SpCRV-1 at 2.6% (2/77), and one sample contained only SYLSV at 1.3% (1/77). Co-infections were detected in 11.7% (9/77) of samples infected with both SCLSDV and SpCRV-1, and 7.8% (6/77) were infected with SCLSDV and SYLSV. SPCLSDV was found in mixed infections, but SpCRV-1 was never found with SYLSV and triple infections involving all three viruses were not observed. The most common virus-like symptoms observed in spiraea included yellowing and chlorotic spotting, mosaic, leaf distortion, and necrotic spots, especially on the edge of the leaves (Fig. 5). These symptoms occurred in single SCLSDV infections in spiraea plants from the same location collected in Minnesota.

### Identification of eriophyid mites

**Fig. 6:**
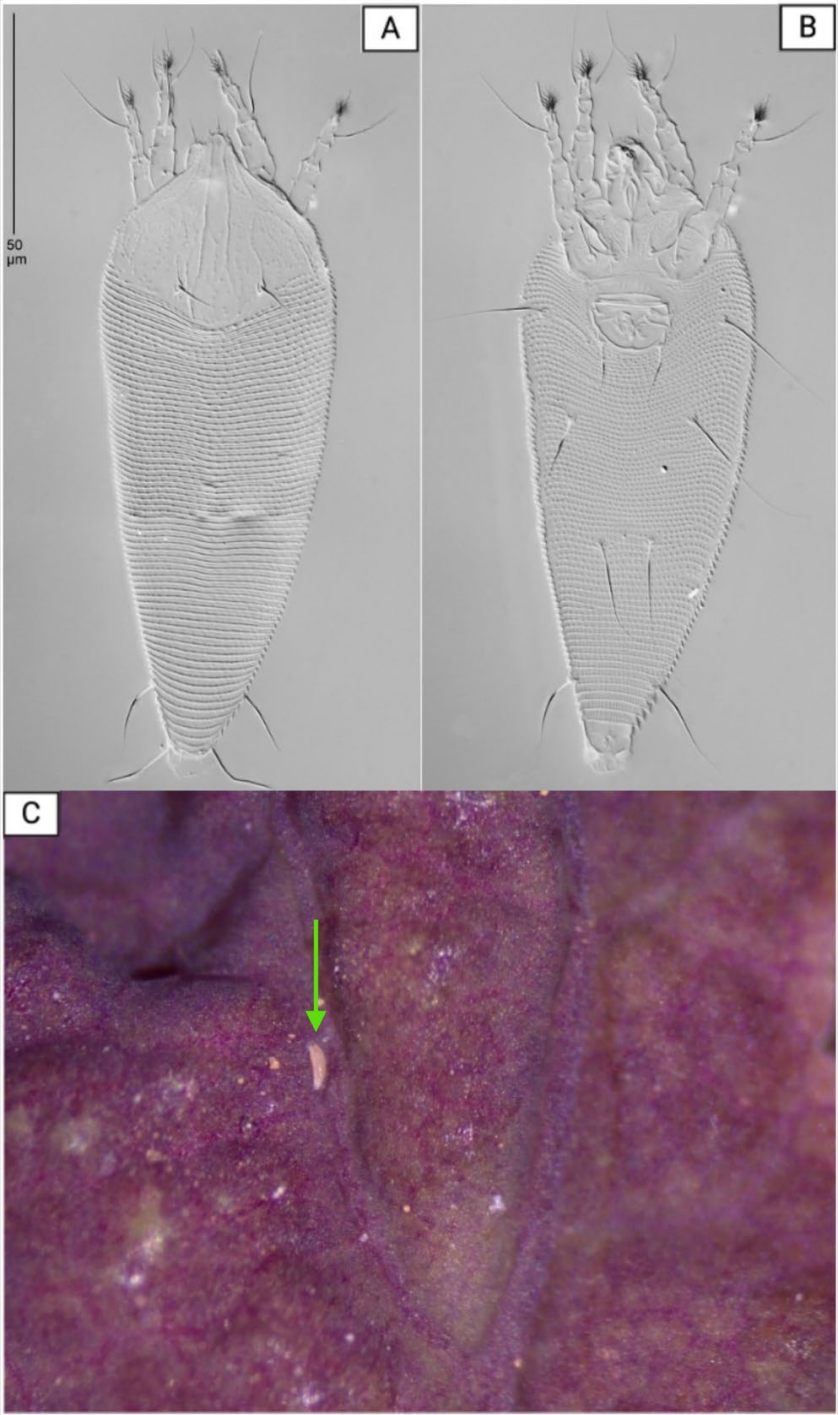
Slide-mounted *Calepitrimerus* mite imaged under a compound microscope: (**A**) dorsal view: (**B**) ventral view: (**C**) mite on the host plant

Eriophyid mites associated with the samples (Fig. 6) were identified as *Calepitrimerus* [3] unambiguously matching all diagnostic character states of the genus, particularly the dorsal opisthosoma bearing one middorsal and two subdorsal ridges, with the middorsal ridge terminating before the subdorsal ridges.

## Discussion

In this study, we report the discovery of two novel viruses infecting spiraea in the USA: Spiraea chlorotic leaf distortion virus (SCLDV; genus *Emaravirus*) and Spiraea alphacytorhabdovirus 1 (SpCRV-1; genus *Alphacytorhabdovirus*). These two viruses were detected by HTS which highlights how next generation sequencing is a great addition to classic virological methods like virion enrichment and TEM, Enzyme-Linked Immunosorbent Assay (ELISA) and Polymerase Chain Reaction (PCR) [2, 32]. Further, SCLDV and SpCRV-1 would not have been detected with routine diagnostic methods like ELISA & PCR [1, 55]. Due to their ability to provide a more comprehensive assessment of viral communities, HTS should greatly improve plant pathogen discovery and diagnostics [8].

The purification of enveloped plant emaraviruses and alphacytorhabdoviruses presents challenges due to their physicochemical characteristics, low titers, and the presence of host secondary metabolites [15, 45]. As a result, these new viruses may have evaded previous detection efforts by virion enrichment and visualization.

Specifically, members of the genus *Emaravirus* are characterized by a lipid bilayer obtained from the host cell membrane, essential for their infectivity and structural stability. PH, ionic composition, temperature, and oxidation of buffers are critical factors to consider during virion enrichment, as they can significantly affect virion stability [14]. Additionally, organic solvents such as ethanol, Triton X-100, n-butanol, and chloroform, which are often employed in purification protocols to remove cellular debris and proteins, may destabilize and dissolve this lipid bilayer and consequently become a challenge for its purification [14]. Once these two viruses were identified by HTS, we addressed these previous issues in our study by modifying an established purification procedure that was originally developed for the tomato spotted wilt virus [53]. By employing a density gradient protocol, we were able to successfully purify and visualize by TEM the intact pleomorphic enveloped particles of SCLSDV. SpCRV-1 was not visualized, which is likely a result of its low infection titer, supported by its low coverage in the HTS results.

The emergence of novel emaraviruses species in recent years has increased the quantity of negative-sense RNA viruses infecting plants. Recently, several unidentified virus-like diseases like rosette disease of roses, the mosaic disease of figs, the sterility mosaic disease of pigeonpea, the ringspot diseases of rowan and oak, and the mosaic disease of aspen trees have been linked to emaraviruses [45]. The discovery and characterization of these viruses remain difficult due to their low abundance, considerable genetic diversity, and technical limitations in enveloped virions visualization and purification. Our research extends the existing knowledge of *Emaravirus* purification techniques and offers potential insights into future molecular and structural analyses of these important diseases.

The genome organization of SCLSDV is similar to those of members of the genus *Emaravirus*; we detected four RNA components corresponding to the core for emaravirus replication. However, additional RNAs had been detected in other emaraviruses, like High Plains wheat mosaic emaravirus with 8 RNA segments and chrysanthemum mosaic-associated virus with 6 RNA [51]. Because the closest relative PCLSaV has 5 RNA segments, we performed a homology-based search using RNA5 of PCLSaV and the 5’ and 3’ conserved termini of SCLSDV, as well as aligning trimmed reads against PCLSaV RNA 5; however, no additional RNAs were detected in SCLSDV.

The genetic diversity of SCLSDV isolates in the USA was further investigated, SCLSDV isolates share nucleotide identity ranged between 77.6% and 95.3%. The putative movement protein was the most conserved suggesting strong functional constraints on viral proteins, whereas the RNA-dependent RNA polymerase was the most divergent, which is consistent with its evolutionary rate in RNA viruses [18, 46]. Phylogenetic analysis showed that all the SCLSDV isolated strains shared a common ancestor.

We also investigated the occurrence of SCLSDV, SpCRV-1, and SYLSV results in symptomatic spiraeas samples suggesting a strong association between yellow spot disease symptoms and SCLSDV infection. SCLSDV’s high prevalence in symptomatic plants indicates its involvement as a principal causal agent. On the other hand, SpCRV-1 was found in lower levels in single and mixed infections, making it a challenge to define its specific role in disease development. Interestingly, across samples collected from a single location, SpCRV-1 indicated the highest positive rate in Oregon (44.4%, *n* = 9). It is unclear if SpCRV-1 is associated with yellow spot disease symptoms since it was only found in 14.3% of total samples and in low titer in the two samples that underwent HTS. Interestingly, plants infected with only SpCRV-1 were asymptomatic. Additional research is needed to identify whether SpCRV-1 is directly associated with symptom development in infected plants. The detection of mixed infections with SCLSDV, SpCRV-1, and SYSLV raises concerns about potential virus-virus interactions. It is unclear how these co-infections affect the intensity of symptoms and progression of disease in spiraea; however, co-infections are known to affect plant-virus interactions and improve pathogenesis in other systems [5].

From an epidemiological perspective, the genetic similarity of virus isolates SCLSDV and SpCRV-1 across the USA suggests the unintentional movement of infected spiraea material facilitated by the propagation of infected stock through cuttings. The detection of SpCRV-1 and SYLSV in multiple states across the USA emphasizes the need for an implementation of stricter monitoring, regular detection surveys, strong sanitation measures, and the use of in vitro culture, meristem culture, and thermotherapy to avoid further spread, especially in commercial nurseries involved in spiraeas propagation and distribution [42, 48, 49]. In this study, RT-PCR primers for detection of both SCLSDV and SpCRV-1 were developed and validated to support further testing and epidemiological studies to prevent virus transmission and mitigate impacts on the ornamental industry.

There are different species of eriophyid mites known to vector viral diseases in the genus *Potyvirus*,* Rymovirus*,* Trichovirus*,* Nepovirus and Emaravirus*: wheat streak mosaic virus (WSMV), ryegrass mosaic virus (RgMV), fig mosaic virus (FMV), cherry mottle leaf virus (CMLV), blackcurrant reversion virus (BRV), pigeonpea sterility mosaic virus 1 and 2 (PPSMV-1, PPSMV-2), wheat mosaic virus (WMoV), raspberry leaf blotch virus (RLBV), and rose rosette virus (RRV), perilla mosaic virus (PerMV), maple mottle associated Virus (MaMaV), High Plains wheat mosaic virus (HPWMoV), European mountain ash ringspot associated virus (EMARAV) [27, 40, 47, 50]. The characterization of virus-transmitting mite species plays an essential role in understanding their ecology and epidemiological significance [54]. Our initial efforts to characterize a potential vector resulted in the identification of mites belonging to the genus *Calepitrimerus* for the first time on spiraea. Although *Calepitrimerus spp*. have not previously been shown to vector plant viruses, another eriophyid mite, *Colomerus vitis*, has been implicated in the transmission of grapevine pino gris virus [35, 54].

U.S. species of *Calepitrimerus* are distinguished by the presence of a pair of spines on the frontal lobe [4]. The most closely related species sharing this character state is *Calepitrimerus achilleae*, which is associated with yarrow (*Achillea millefolium* L.). However, *C. achilleae* differs by having multiple small denticles on the frontal lobe. This study did not confirm the transmission by eriophyid mites as reported for other members of the genus *Emaravirus*. However, eriophyid mites were frequently associated with symptomatic plant samples. Further work should focus on transmission experiments to determine whether *Calepitirmerus* can act as a virus vector, as well as molecular detection of the virus in the mites associated with infected spiraea plants. Other additional studies on mite ecology, feeding behavior, and host range would help to determine its potential role in virus epidemiology.

## Supplementary Information

Below is the link to the electronic supplementary material.

Supplementary Fig. S1 Panhandle structures assembled from 5’ and 3’ termini of Spiraea chlorotic leaf spot distortion virus (SCLSDV) RNAs.

Supplementary Fig. S2 Read coverage for the four RNA segments of Spiraea chlorotic leaf spot distortion virus (SCLSDV). Plots display sequencing read depth per nucleotide position in different segments: A, RNA 1- RdRP; B, RNA 2 - GP; C, RNA 3 - NC; D, RNA 4 - MP. The nucleotide position is represented by the x-axis, while the number of reads mapped to each position is represented by the y-axis.

Supplementary Fig. S3. Multiple alignments of conserved amino acid motif detected of the RdRp: (A) emaravirus; (B) alphacytorhabdovirus.

Supplementary Fig. S4 N’ terminal endonuclease domains of RdRps in Spiraea chlorotic leaf spot distortion virus (SCLSDV) and the closest related emaraviruses. Multiple sequence alignment was generated using Clustal Omega in Geneious prime 2025.0.3.

Supplementary Fig. S5 Amino acid stretches of RNA3 in Spiraea chlorotic leaf spot distortion virus (SCLSDV). Multiple sequence alignment was generated using Clustal Omega in Geneious prime 2025.0.3.

Supplementary Fig. S6 Read coverage of the two genomes of Spiraea alphacytorhabdovirus 1 (SpCRV-1): A, SP2019; B, SPR-OR6. The nucleotide position is represented by the x-axis, while the number of reads mapped to each position is represented by the y-axis.

Supplementary Fig. S7 Alignments of conserved intergenic regions located downstream of each ORF of Spiraea alphacytorhabdovirus 1 (SpCRV-1) are shown in green boxes;, grey boxes comprise poly-U tract (element I), intergenic spacers (element II), and the putative transcription initiation sequence of the following gene (element III).

Supplementary Table S1 Summary of the 77 samples with symptomatic and asymptomatic Spiraea plants and tested for Spiraea chlorotic leaf spot distortion virus (SCLSDV) (genus *Emaravirus*), Spiraea alphacytorhabdovirus 1 (SpCRV-1) (genus *Alphacytorhabdovirus)*, and Spirea yellow leaf spot virus (SYLSV) (genus *Badnavirus*).

Supplementary Table S2 List of primers used to amplify RNA 5’ and 3’ termini by RACE amplification.

Supplementary Table S3 Summary of sequence features of Spiraea alphacytorhabdovirus 1 (SpCRV-1).

Supplementary Table S4 Amino acid pairwise comparisons (%) between hypothetical proteins from Spiraea alphacytorhabdovirus 1 (SpCRV-1) and its closest relatives.
