# Supplementary figures and images for: Discovery of a novel emaravirus and an alphacytorhabdovirus infecting Spiraea in the USA
Source: Arch Virol. 2026 Jun 11;171(7):205. doi: 10.1007/s00705-026-06640-2 (PMC13253887; doi:10.1007/s00705-026-06640-2)

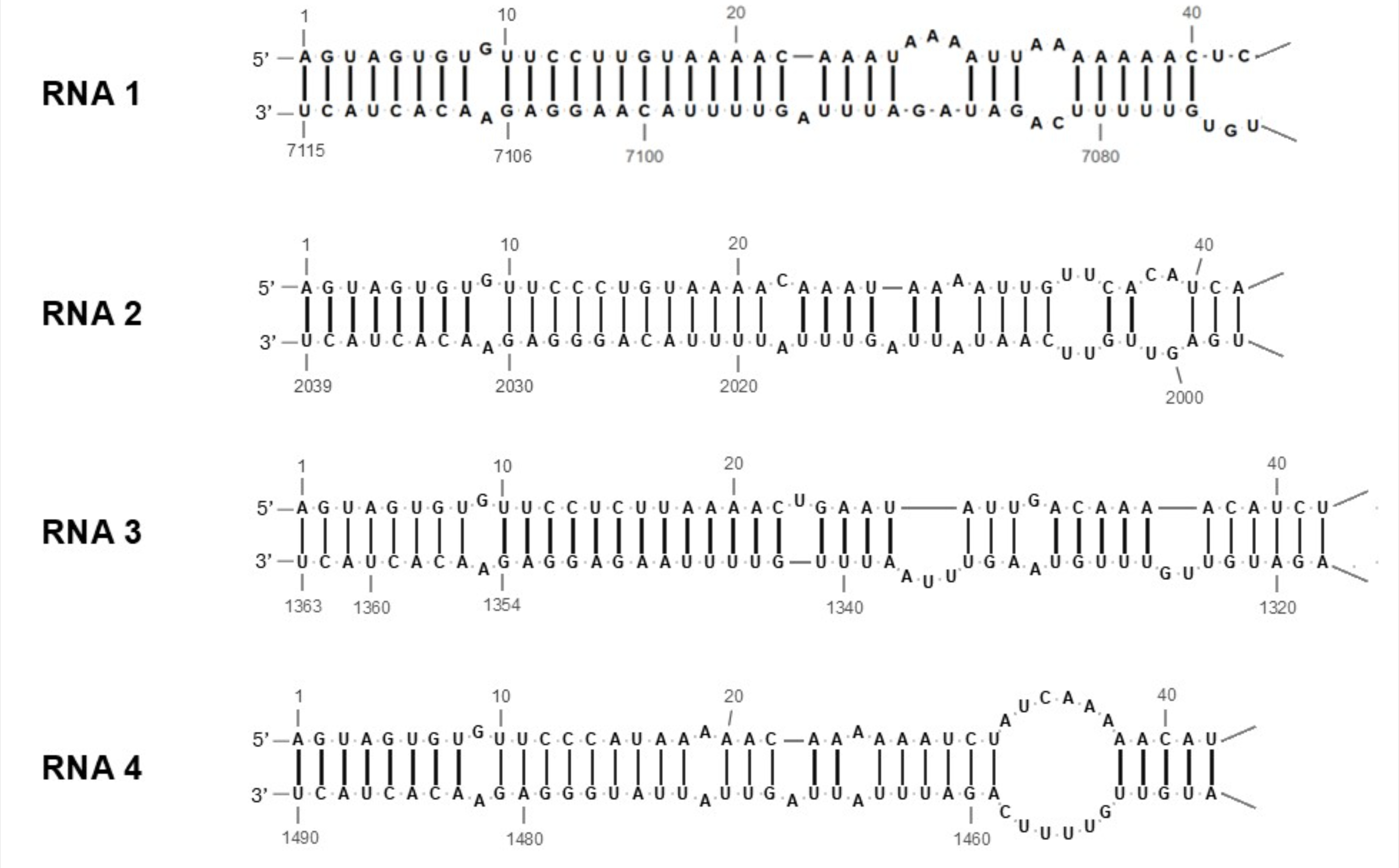

Supplement: Supplementary file 1 — Supplementary Fig. S1 Panhandle structures assembled from 5’ and 3’ termini of Spiraea chlorotic leaf spot distortion virus (SCLSDV) RNAs. [file 705_2026_6640_MOESM1_ESM.jpeg]

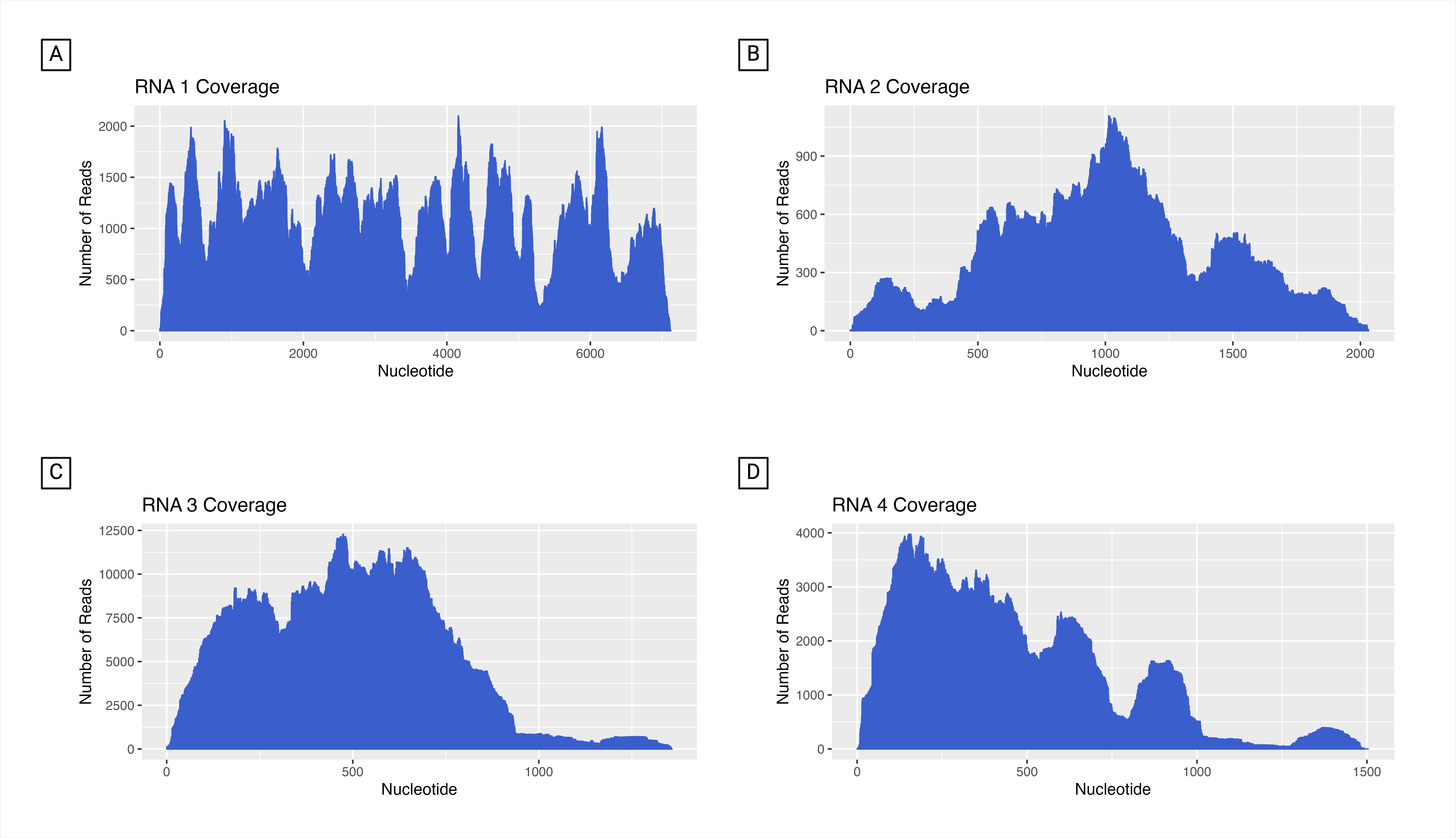

Supplement: Supplementary file 2 — Supplementary Fig. S2 Read coverage for the four RNA segments of Spiraea chlorotic leaf spot distortion virus (SCLSDV). Plots display sequencing read depth per nucleotide position in different segments: A, RNA 1- RdRP; B, RNA 2 - GP; C, RNA 3 - NC; D, RNA 4 - MP. The nucleotide position is represented by the x-axis, while the number of reads mapped to each position is represented by the y-axis. [file 705_2026_6640_MOESM2_ESM.jpeg]

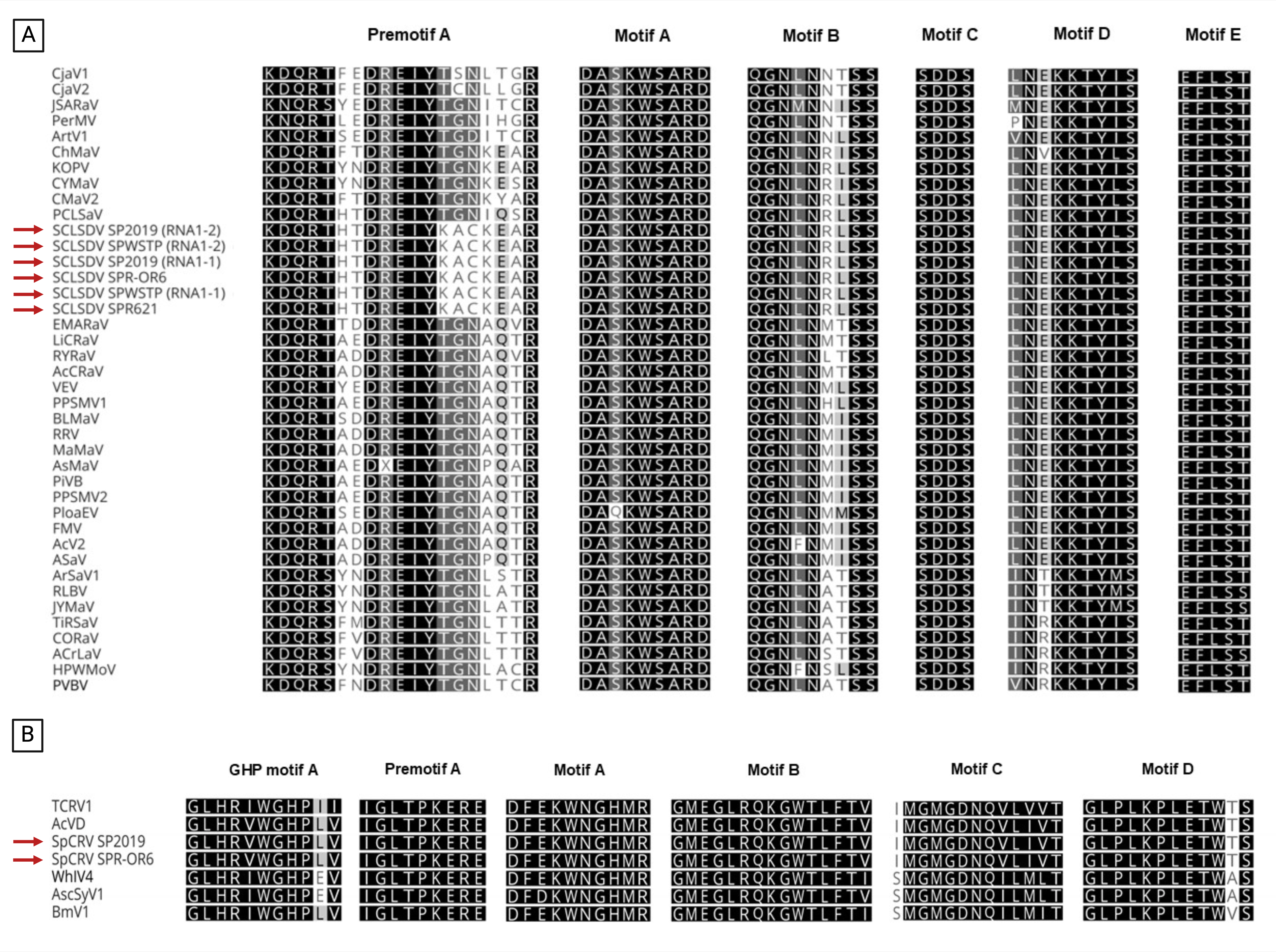

Supplement: Supplementary file 3 — Supplementary Fig. S3. Multiple alignments of conserved amino acid motif detected of the RdRp: (A) emaravirus; (B) alphacytorhabdovirus. [file 705_2026_6640_MOESM3_ESM.jpeg]

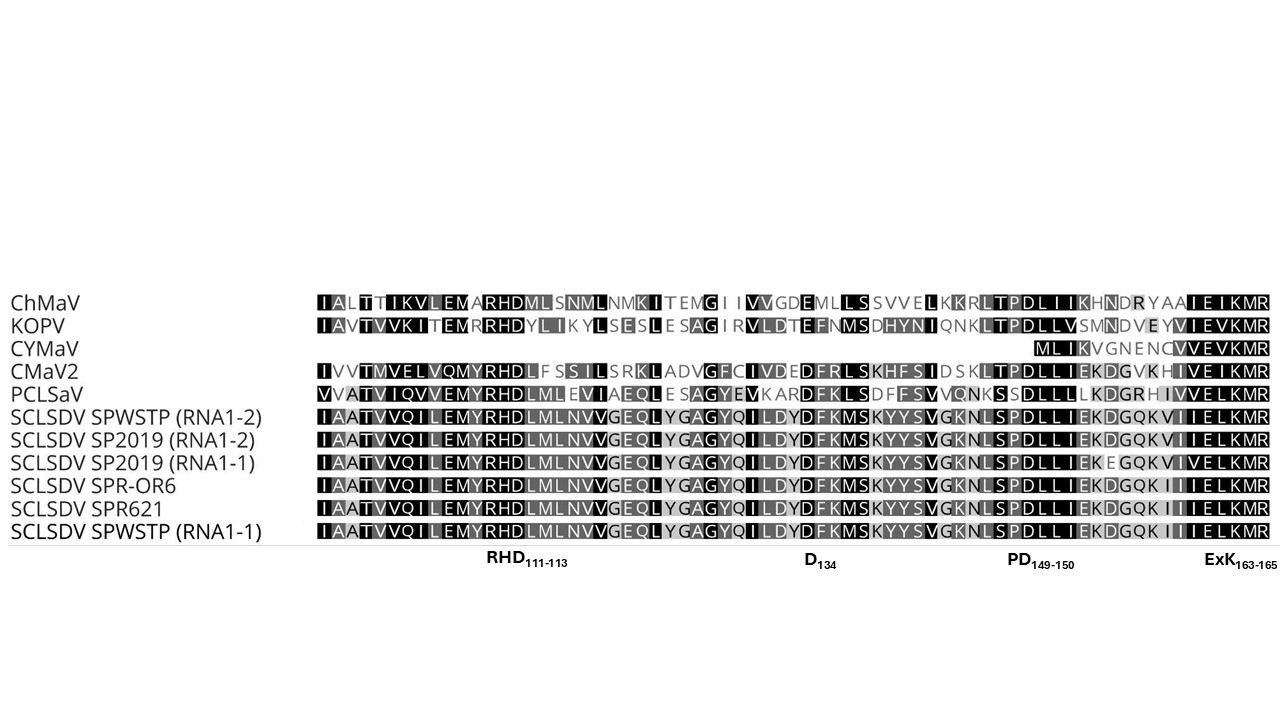

Supplement: Supplementary file 4 — Supplementary Fig. S4 N’ terminal endonuclease domains of RdRps in Spiraea chlorotic leaf spot distortion virus (SCLSDV) and the closest related emaraviruses. Multiple sequence alignment was generated using Clustal Omega in Geneious prime 2025.0.3. [file 705_2026_6640_MOESM4_ESM.jpg]

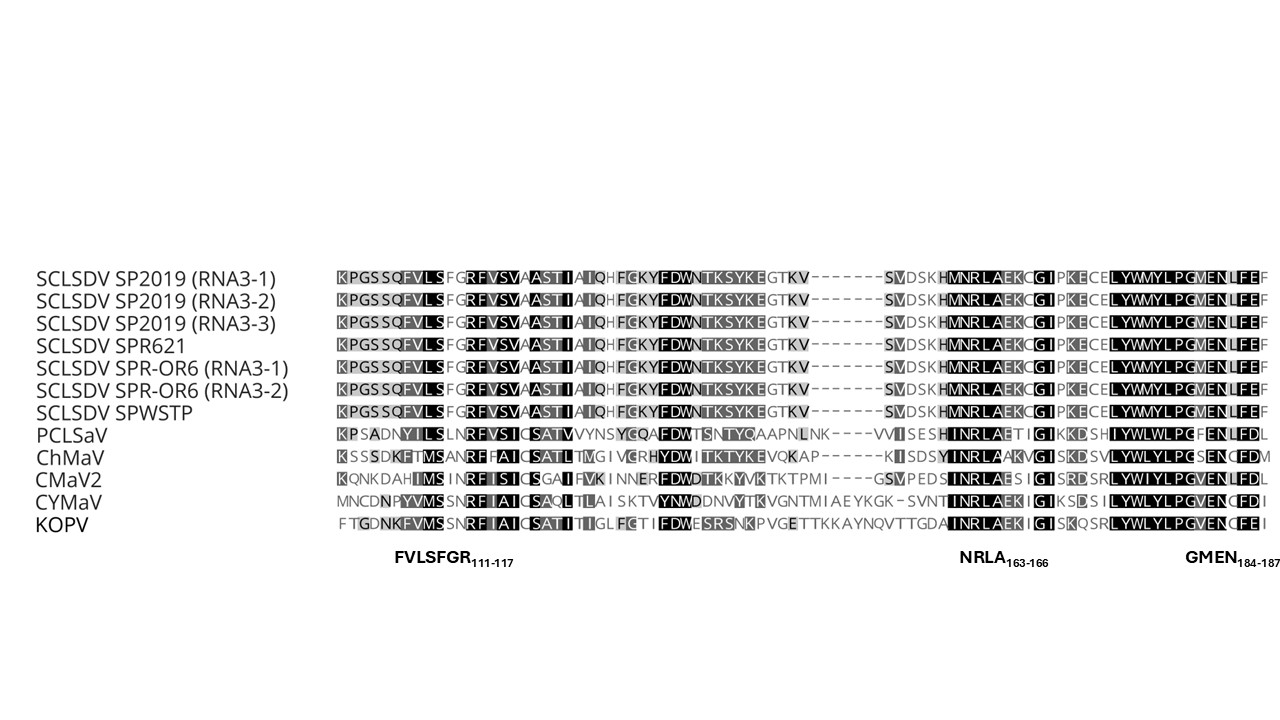

Supplement: Supplementary file 5 — Supplementary Fig. S5 Amino acid stretches of RNA3 in Spiraea chlorotic leaf spot distortion virus (SCLSDV). Multiple sequence alignment was generated using Clustal Omega in Geneious prime 2025.0.3. [file 705_2026_6640_MOESM5_ESM.jpg]

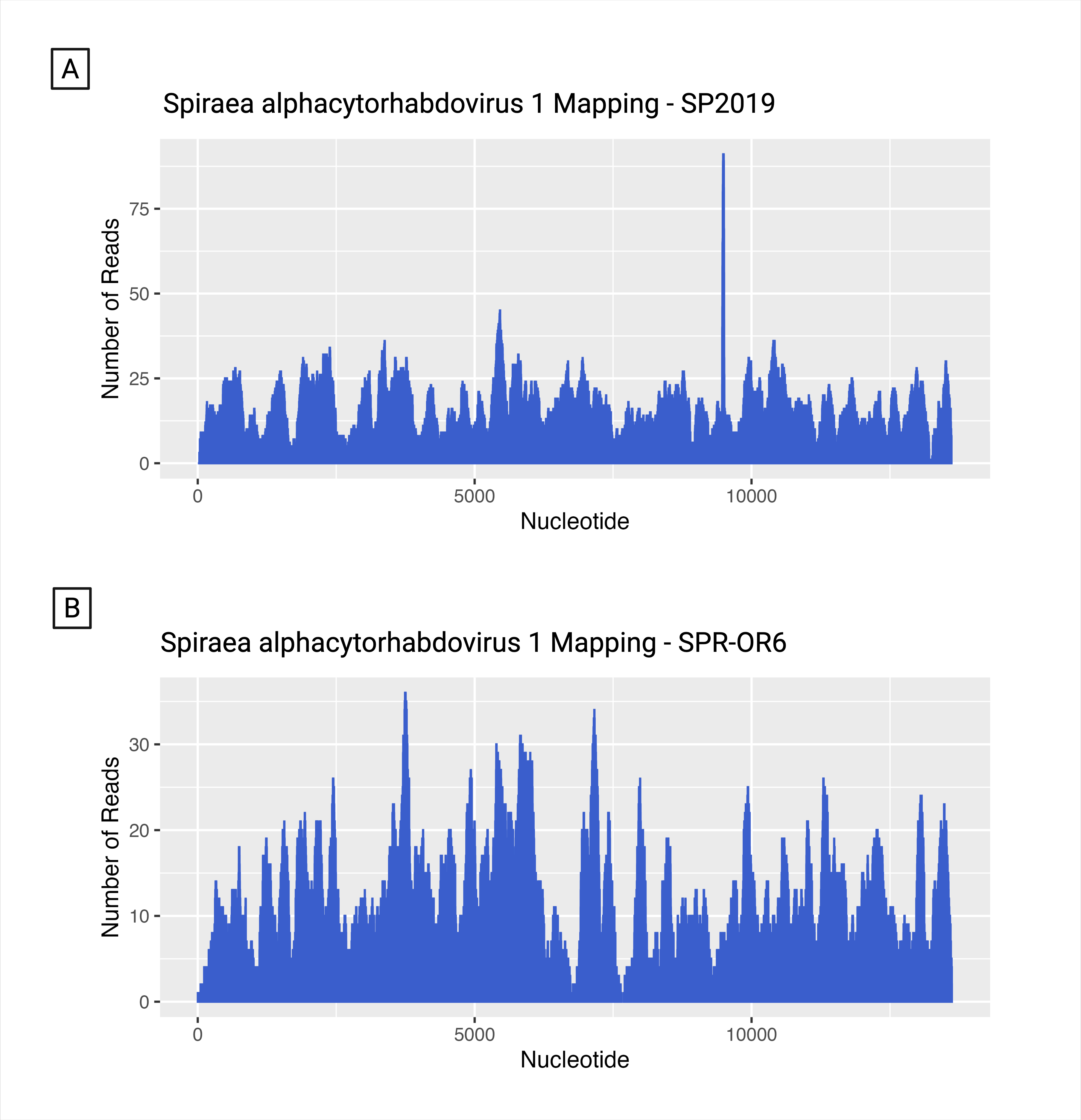

Supplement: Supplementary file 6 — Supplementary Fig. S6 Read coverage of the two genomes of Spiraea alphacytorhabdovirus 1 (SpCRV-1): A, SP2019; B, SPR-OR6. The nucleotide position is represented by the x-axis, while the number of reads mapped to each position is represented by the y-axis. [file 705_2026_6640_MOESM6_ESM.jpeg]

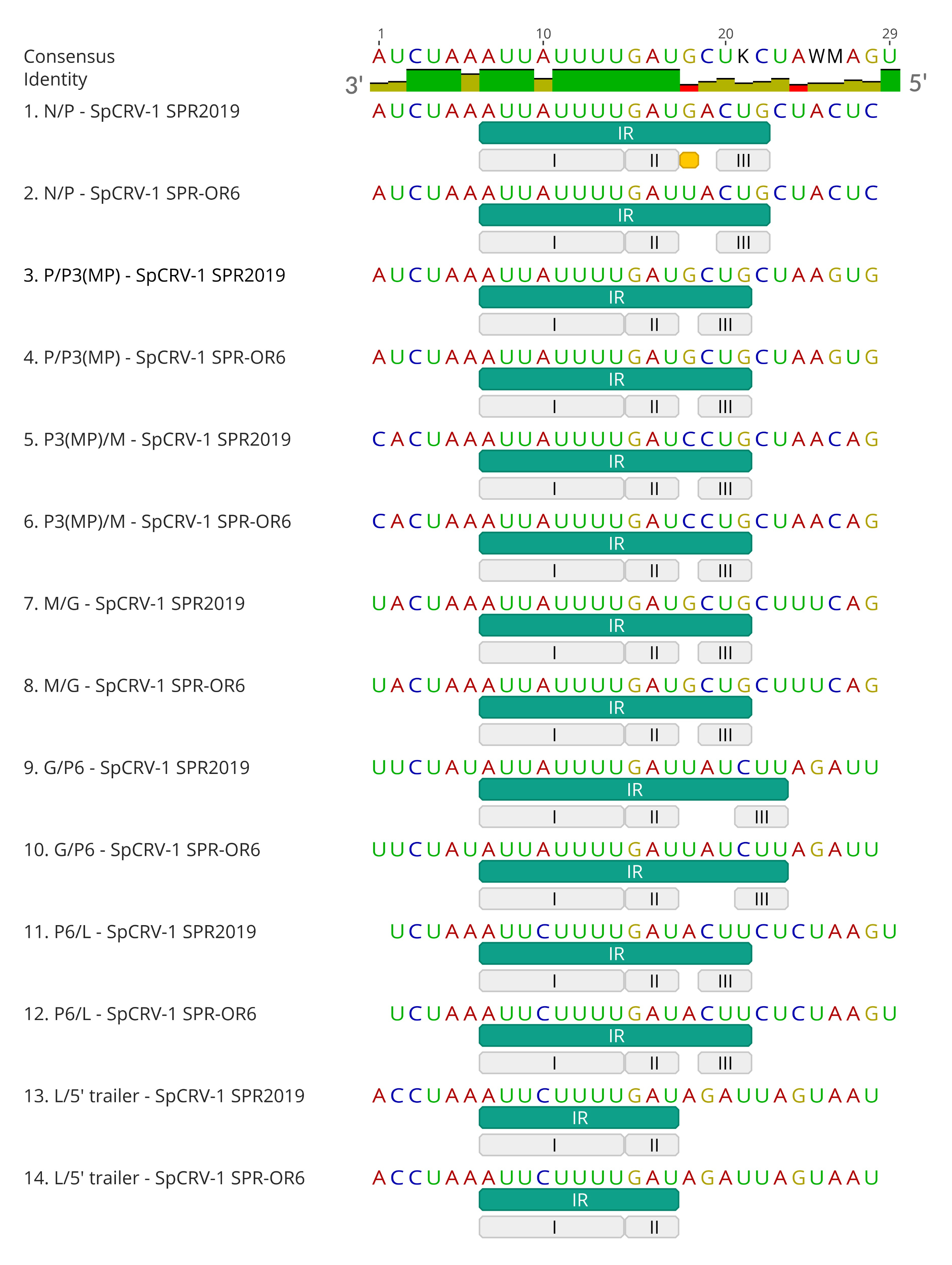

Supplement: Supplementary file 7 — Supplementary Fig. S7 Alignments of conserved intergenic regions located downstream of each ORF of Spiraea alphacytorhabdovirus 1 (SpCRV-1) are shown in green boxes;, grey boxes comprise poly-U tract (element I), intergenic spacers (element II), and the putative transcription initiation sequence of the following gene (element III). [file 705_2026_6640_MOESM7_ESM.jpeg]
