# Supplementary material for: Discovery of a novel emaravirus and an alphacytorhabdovirus infecting Spiraea in the USA
Source: Arch Virol. 2026 Jun 11;171(7):205. doi: 10.1007/s00705-026-06640-2 (PMC13253887; doi:10.1007/s00705-026-06640-2)
Supplement: Supplementary file 8 — Supplementary Table S1 Summary of the 77 samples with symptomatic and asymptomatic Spiraea plants and tested for Spiraea chlorotic leaf spot distortion virus (SCLSDV) (genus Emaravirus), Spiraea alphacytorhabdovirus 1 (SpCRV-1) (genus Alphacytorhabdovirus), and Spirea yellow leaf spot virus (SYLSV) (genus Badnavirus). [file 705_2026_6640_MOESM8_ESM.docx]

Supplementary Table S1 Summary of the 77 samples with symptomatic and asymptomatic Spiraea plants and tested for Spiraea chlorotic leaf spot distortion virus (SCLSDV) (genus *Emaravirus*), Spiraea alphacytorhabdovirus 1 (SpCRV-1) (genus *Alphacytorhabdovirus*), and Spirea yellow leaf spot virus (SYLSV) (genus *Badnavirus*).

| **Sample No.** | **Collection Year** | **Location** ^a^ | **Species - Cultivar** | **Viral Infection** ^b^ | | | **Symptoms** ^c^ |
| --- | --- | --- | --- | --- | --- | --- | --- |
|  |  |  |  | **SCLSDV** | **SpCRV-1** | **SYLSV** |  |
| 1 | 2017 | MN | Spirea spp. | Positive | Negative | Positive | N/A |
| 2 | 2018 | PDC | Spirea spp. | Positive | Negative | Negative | N/A |
| 3 | 2019 | MN | Spiraea arboricola | Positive | Negative | Negative | N/A |
| 4 | 2019 | MN | Spiraea japonica - Neon Flash | Positive | Positive | Negative | N/A |
| 5 | 2019 | MN | Spiraea japonica - Spiraea x bumalda Anthony Waterer | Positive | Negative | Negative | N/A |
| 6 | 2019 | MN | Spiraea betulifolia - Tor Birch leaf | Positive | Negative | Negative | N/A |
| 7 | 2019 | MN | Spiraea japonica - Spiraea x bumalda Anthony Waterer | Negative | Negative | Negative | N/A |
| 8 | 2019 | MN | Spiraea japonica - Gold Flame | Positive | Negative | Positive | N/A |
| 9 | 2019 | MN | Spiraea japonica - Gold mount | Positive | Negative | Positive | N/A |
| 10 | 2019 | MN | Spiraea japonica - Walbuma (Magic Carpet) | Positive | Negative | Negative | N/A |
| 11 | 2019 | MN | Spiraea japonica - Spiraea x bumalda Anthony Waterer | Positive | Positive | Negative | N/A |
| 12 | 2019 | MN | Spiraea japonica - Neon Flash | Positive | Positive | Negative | N/A |
| 13 | 2019 | MN | Spiraea albiflora | Positive | Positive | Negative | N/A |
| 14 | 2019 | MN | Spiraea japonica - Double Play candy corn | Positive | Negative | Positive | N/A |
| 15 | 2019 | MN | Spiraea japonica - Golden Elf | Positive | Negative | Positive | N/A |
| 16 | 2019 | MN | Spirea spp. | Positive | Negative | Negative | N/A |
| 17 | 2019 | MN | Spirea spp. | Positive | Negative | Negative | N/A |
| 18 | 2019 | MN | Spirea spp. | Negative | Negative | Positive | N/A |
| 19 | 2019 | MN | Spirea spp. | Positive | Negative | Negative | N/A |
| 20 | 2019 | MN | Spirea spp. | Positive | Negative | Positive | N/A |
| 21 | 2019 | MN | Spirea spp. | Positive | Negative | Negative | N/A |
| 22 | 2019 | MN | Spirea spp. | Positive | Negative | Negative | N/A |
| 23 | 2019 | MN | Spirea spp. | Positive | Negative | Negative | N/A |
| 24 | 2019 | MN | Spirea spp. | Positive | Negative | Negative | N/A |
| 25 | 2019 | PDC | Spirea spp. | - | Negative | Negative | N/A |
| 26 | 2019 | PDC | Spirea spp. | Positive | Negative | Negative | N/A |
| 27 | 2019 | PDC | Spirea spp. | Positive | Negative | Negative | N/A |
| 28 | 2019 | PDC | Spirea spp. | Positive | Negative | Negative | N/A |
| 29 | 2024 | MN | Spirea spp. | Positive | Negative | Negative | N/A |
| 30 | 2024 | MN | Spirea spp. | Positive | Negative | Negative | N/A |
| 31 | 2024 | MN | Spirea spp. | Positive | Negative | Negative | N/A |
| 32 | 2024 | MN | Spiraea japonica - Double Play | Positive | Negative | Negative | Symptoms |
| 33 | 2024 | MN | Spiraea japonica - Double Play | Positive | Negative | Negative | Symptoms |
| 34 | 2024 | MN | Spiraea japonica - Gold Mound | Positive | Negative | Negative | Symptoms |
| 35 | 2024 | MN | Spiraea japonica - Gold Mound | Positive | Negative | Negative | Symptoms |
| 36 | 2024 | MN | Spiraea japonica - Carpet pink | Negative | Negative | Negative | N/A |
| 37 | 2024 | MN | Spiraea japonica - Carpet pink | Negative | Negative | Negative | N/A |
| 38 | 2024 | MN | Spiraea japonica - Pink | Negative | Negative | Negative | N/A |
| 39 | 2024 | MN | Spiraea japonica - Pink | Negative | Negative | Negative | N/A |
| 40 | 2024 | MN | Spiraea japonica - Carpet pink | Positive | Negative | Negative | Symptoms |
| 41 | 2024 | MN | Spiraea japonica - Carpet pink | Positive | Negative | Negative | Symptoms |
| 42 | 2024 | MN | Spirea spp. | Positive | Positive | Negative | N/A |
| 43 | 2024 | MN | Spiraea japonica - Double Play Blue Kazoo | Negative | Negative | Negative | N/A |
| 44 | 2024 | MN | Spiraea japonica | Positive | Negative | Negative | Symptoms |
| 45 | 2024 | MN | Spiraea japonica - Neon Flash | Positive | Positive | Negative | Symptoms |
| 46 | 2024 | MN | Spiraea japonica - Little princess | Positive | Negative | Negative | Symptoms |
| 47 | 2024 | MN | Spiraea japonica - Little Spark | Positive | Negative | Negative | Symptoms |
| 48 | 2024 | MN | Spiraea japonica - Magic Carpet | Positive | Negative | Negative | Symptoms |
| 49 | 2024 | PDC | Spirea spp. | - | Negative | Negative | N/A |
| 50 | 2024 | PDC | Spiraea japonica - Little Spark | Negative | Positive | Negative | N/A |
| 51 | 2024 | PDC | Spiraea japonica - Gold Mound | Negative | Negative | Negative | N/A |
| 52 | 2024 | PDC | Spiraea japonica - Gold Mound | Negative | Negative | Negative | N/A |
| 53 | 2024 | PDC | Spiraea japonica - Gold Mound | Negative | Negative | Negative | N/A |
| 54 | 2024 | PDC | Spirea spp. | Negative | Negative | Negative | N/A |
| 55 | 2024 | PDC | Spiraea japonica - Magic Carpet | Negative | Negative | Negative | N/A |
| 56 | 2024 | PDC | Spiraea japonica - Magic Carpet | Negative | Negative | Negative | N/A |
| 57 | 2024 | PDC | Spiraea albiflora - Japanese White | Positive | Negative | Negative | N/A |
| 58 | 2024 | PDC | Spiraea japonica - Gold Flame | Positive | Negative | Negative | N/A |
| 59 | 2024 | PDC | Spirea spp. | Negative | Negative | Negative | N/A |
| 60 | 2024 | OR | Spiraea albiflora - Japanese White | Positive | Positive | Negative | Symptoms |
| 61 | 2024 | OR | Spiraea albiflora - Japanese White | Positive | Positive | Negative | Symptoms |
| 62 | 2024 | OR | Spiraea japonica - Spiraea x bumalda Anthony Waterer | Positive | Negative | Negative | Symptoms |
| 63 | 2024 | OR | Spiraea japonica - Spiraea x bumalda Anthony Waterer | Positive | Negative | Negative | Symptoms |
| 64 | 2024 | OR | Spiraea japonica - Neon Flash x Cherry Red | Positive | Negative | Negative | Symptoms |
| 65 | 2024 | OR | Spiraea japonica - Neon Flash x Cherry Red | Positive | Positive | Negative | Symptoms |
| 66 | 2024 | OR | Spiraea japonica - Neon Flash | Negative | Negative | Negative | N/A |
| 67 | 2024 | OR | Spiraea japonica - Spiraea x bumalda Anthony Waterer | Negative | Positive | Negative | N/A |
| 68 | 2024 | OR | Spiraea albiflora | Negative | Negative | Negative | N/A |
| 69 | 2024 | NY | Spiraea japonica - Double Play | Negative | Negative | Negative | N/A |
| 70 | 2024 | NY | Spiraea japonica - Double Play | Negative | Negative | Negative | N/A |
| 71 | 2024 | NY | Spiraea blumalda - Gold flake | Negative | Negative | Negative | N/A |
| 72 | 2024 | NY | Spiraea japonica - Little princess | Negative | Negative | Negative | N/A |
| 73 | 2024 | NY | Spirea media - Spiraea Blue Kazoo | Negative | Negative | Negative | N/A |
| 74 | 2024 | NY | Spiraea japonica - Double Play | Negative | Negative | Negative | N/A |
| 75 | 2024 | NY | Spiraea nipponica - Snowmound | Negative | Negative | Negative | N/A |
| 76 | 2024 | OH | Spirea spp. | Positive | Negative | Negative | N/A |
| 77 | 2024 | IN | Spirea spp. | Positive | Negative | Negative | N/A |

^a^ From Minnesota, MN; New York, NY; Ohio, OH; Indiana, IN; and received in Plant Disease Clinic, PDC, in Minnesota.

^b^ Viral infection determined by RT-PCR. SCLSDV: Spiraea chlorotic leaf spot distortion virus, SpCRV-1: Spiraea cytorhabdovirus 1, SYLSV: Spiraea yellow leaf spot virus.

^c^ N/A unavailable data
