# Supplementary material for: Discovery of a novel emaravirus and an alphacytorhabdovirus infecting Spiraea in the USA
Source: Arch Virol. 2026 Jun 11;171(7):205. doi: 10.1007/s00705-026-06640-2 (PMC13253887; doi:10.1007/s00705-026-06640-2)
Supplement: Supplementary file 9 — Supplementary Table S2 List of primers used to amplify RNA 5’ and 3’ termini by RACE amplification. [file 705_2026_6640_MOESM9_ESM.docx]

Supplementary Table S2 List of primers used to amplify RNA 5’ and 3’ termini by RACE amplification.

| **RNA**^a^ | **Primer name**^b^ | **Sequence (5' - 3')** | **Position (nt)**^c^ | **Length (bp)** |
| --- | --- | --- | --- | --- |
| RNA 1 | SP_EmV_R1_5endF1 | GCACACAACACAATAGAAGATGC | 5954-5976 | 1162 |
|  | SP_EmV_R1_5endF2 | ACATATTATTCACCATTCAACTCTGT | 6842-6867 | 274 |
|  | SP_EmV_R1_3endR1 | GCATCAAGTCATGCCGATACATCTCC | 376-401 | 401 |
|  | SP_EmV_R1_3endR2 | TCTCTCTTAGTGAAATCAATGTTGTC | 260-285 | 285 |
|  | 5H-SPEMV-R | **CTCAGC**AGTAGTGTTCTCC | 5’ end | - |
| RNA 2 | SP_EmV_R2_5endF3 | AGTCTCATATCACAGACCCTATAGTTG | 1701-1727 | 339 |
|  | SP_EmV_R2_5endF4 | GTCTCAGGATGCTACAACTGCCATCAA | 1460-1486 | 580 |
|  | SP_EmV_R2_3endR1 | GCACATATGACATGACATGATTGAC | 410-434 | 434 |
|  | SP_EmV_R2_3endR2 | CAAATGCACAGCTGCATGTTGAGC | 159-182 | 182 |
|  | 5H-SPEMV-R | **CTCAGC**AGTAGTGTTCTCC | 5’ end | - |
| RNA 3 | SP_EmV_R3_5endF3 | CTGTCTAACAAGATGGTGAAGAAAGGC | 769-795 | 580 |
|  | SP_EmV_R3_5endF4 | GTGTGAGCTGTATTGGATGTATCTGC | 612-637 | 752 |
|  | SP_EmV_R3_3endR1 | CTGCCAACCTGTTCATGTGCTTGC | 566-589 | 589 |
|  | SP_EmV_R3_3endR2 | CTGCACTTGCATCAGGAATGCTGTC | 196-220 | 220 |
|  | 5H-SPEMV-R | **CTCAGC**AGTAGTGTTCTCC | 5’ end | - |
| RNA 4 | SP_EmV_R4_5endF1 | CAGGAAAGGCAAGAGTGATGGTAGC | 430-454 | 1061 |
|  | SP_EmV_R4_5endF2 | AGAAGGCTATAGCAGACACCAAGG | 951-974 | 540 |
|  | SP_EmV_R4_3endR1 | GCTCTGGTAGGTCTTCTATTGGC | 679-701 | 701 |
|  | SP_EmV_4_3endR2 | CCCAGAGTTTCACATCATGCTCAGC | 152-176 | 176 |
|  | 5H-SPEMV-R | **CTCAGC**AGTAGTGTTCTCC | 5’ end | - |
| 3' RACE | Oligo dT-anchor primer (vial 8)^d^ | GACCACGCGTATCGATGTCGACTTTTTTTTTTTTTTTTV | - | - |
|  | PCR Anchor (vial 9)^d^ | GACCACGCGTATCGATGTCGAC | - | - |

^a^ RACE = rapid amplification of cDNA ends

^b^ Primers were designed determine the termini regions of the viral RNA1 to RNA4; primer 5H-SPEMV-R was designed based on the conserved terminal sequence of Spiraea chlorotic leaf spot distortion virus (SCLSDV): and bold nucleotides marked sequences of no viral origin (Zheng et al. 2017).

^c^ Targeted positions of primers were referred from SCLSDV genome RNAs; nt = nucleotide.

^d^ Oligo dT-anchor primer (vial 8) and PCR Anchor (vial 9) from 5′/3′ RACE Kit 2nd Generation Roche kit
