# Supplementary material for: Discovery of a novel emaravirus and an alphacytorhabdovirus infecting Spiraea in the USA
Source: Arch Virol. 2026 Jun 11;171(7):205. doi: 10.1007/s00705-026-06640-2 (PMC13253887; doi:10.1007/s00705-026-06640-2)
Supplement: Supplementary file 10 — Supplementary Table S3 Summary of sequence features of Spiraea alphacytorhabdovirus 1 (SpCRV-1). [file 705_2026_6640_MOESM10_ESM.docx]

Supplementary Table S3 Summary of sequence features of Spiraea alphacytorhabdovirus 1 (SpCRV-1).

| **ORF number**^a^ | **Protein**^b^ | **Protein size (aa)**^c^ | **Calculated MW (kDa)**^d^ | **Putative function**^e^ | **TM**^f^ | **NLS (Score)**^g^ |
| --- | --- | --- | --- | --- | --- | --- |
| 1 | N | 482 | 54.05 | Nucleocapsid protein | None | **NLS (Score)**^g^ |
| 2 | P’ | 51 | 6.28 | Unknown | Phobius: 6-25 aa  SMART: 13-35 aa | Bipartite (5.3)  Sequence: EVNAMKLLFNAEKGRKNKDKGETSQTAKNSKEE_8-51_ |
| 3 | P | 304 | 34.33 | Phosphoprotein | None | None |
| 4 | P3 (MP) | 365 | 40.40 | Movement protein | None | None |
| 5 | M | 189 | 20.89 | Matrix protein | None | Bipartite (4.2)  Sequence: TLSKKKYYLDEEIIMGAIEKKRKEY_336-360_ |
| 6 | G | 551 | 62.53 | Glycoprotein | 506-528 aa | Bipartite (3.1) |
| 7 | P6 | 61 | 6.96 | Unknown | 15-37 aa | None |
| 8 | L | 2122 | 243.09 | RNA- dependent RNA polymerase | None | Bipartite (2.1) |

^a^ Number of predicted open reading frames (ORFs).

^b^ Encoded proteins including nucleocapsid (N), an unknown protein (P’), phosphoprotein (P), movement protein (P3/MP), matrix protein (M), glycoprotein (G), an unknown protein (P6), and RNA-dependent RNA polymerase (L).

^c^ Protein size (aa: amino acids).

^d^ calculated molecular weight (MW) in kilodaltons (kDa).

^e^ Putative function.

^f^ Predicted transmembrane domains (TM).

^g^ NLS: nuclear localization signal with scores provided.
