# Supplementary material for: Discovery of a novel emaravirus and an alphacytorhabdovirus infecting Spiraea in the USA
Source: Arch Virol. 2026 Jun 11;171(7):205. doi: 10.1007/s00705-026-06640-2 (PMC13253887; doi:10.1007/s00705-026-06640-2)
Supplement: Supplementary file 11 — Supplementary Table S4 Amino acid pairwise comparisons (%) between hypothetical proteins from Spiraea alphacytorhabdovirus 1 (SpCRV-1) and its closest relatives. [file 705_2026_6640_MOESM11_ESM.docx]

Supplementary Table S4 Amino acid pairwise comparisons (%) between hypothetical proteins from Spiraea alphacytorhabdovirus 1 (SpCRV-1) and its closest relatives.

| **SpCRV-1 versus** | **Nucleocapsid (N)** | **Phosphoprotein (P)** | **P3(MP)** | **Matrix (M)** | **Glycoprotein (G)** | **P6** | **Polymerase (L)** |
| --- | --- | --- | --- | --- | --- | --- | --- |
| AcVD | 82.8 | 80.9 | 87.6 | 79.4 | 79.5 | 22.6 | 83.8 |
| IleACRV1 | 56.2 | 38.5 | 51.8 | 37.3 | 49.2 | 24.2 | 58.4 |
| CynACRV1 | 47.4 | 34.0 | 50.5 | 37.0 | 41.4 | - | 52.8 |
| TCRV1 | 49.0 | 32.7 | 51.1 | 13.2 | 41.8 | 20.0 | 53.5 |
| HaCV | 39.5 | 26.5 | 34.7 | 24.3 | 32.9 | 11.1 | 45.6 |
| PinACRV1 | 38.8 | 24.1 | 35.1 | 18.7 | 31.1 | 19.0 | 45.9 |
| PelRaV1 | 47.9 | 31.4 | 50.4 | 37.3 | - | - | - |
| LotCorV1 | 50.2 | 31.5 | 52.0 | 33.9 | 42.8 | 13.1 | 50.0 |

Notes: The table presents sequence identity percentages of pairwise comparisons for seven predicted proteins using Clustal Omega; nucleocapsid (N), phosphoprotein (P), movement protein (P3/MP), matrix protein (M), glycoprotein (G), protein P6, and RNA-dependent RNA polymerase (L).

Virus abbreviations: Actinidia virus D (AcVD), Ilex alphacytorhabdovirus 1 (IleACRV1), Cynara alphacytorhabdovirus 1 (CynACRV1), Taraxacum cytorhabdovirus 1 (TCRV1), Honeysuckle-associated cytorhabdovirus 1 (HaCV), Pinellia alphacytorhabdovirus 1 (PinACRV1), Pelargonium radula virus 1 (PelRaV1), and Lotus corniculatus virus 1 (LotCorV1). Dashes indicate the absence of a coding sequence in the genome of the virus.
